# *In vitro* potency, *in vitro* and *in vivo* efficacy of liposomal alendronate in combination with γδ T cell immunotherapy in mice

**DOI:** 10.1016/j.jconrel.2016.09.023

**Published:** 2016-11-10

**Authors:** Naomi O. Hodgins, Wafa' T. Al-Jamal, Julie T-W. Wang, Ana C. Parente-Pereira, Mao Liu, John Maher, Khuloud T. Al-Jamal

**Affiliations:** a King's College London, 150 Stamford Street, Institute of Pharmaceutical Science, Franklin-Wilkins Building, 150 Stamford Street, London SE1 9NH, UK; b School of Pharmacy, University of East Anglia, Norwich Research Park, Norwich NR4 7TJ, UK; c King's College London, Division of Cancer Studies, Guy's Hospital, London SE1 9RT, UK

**Keywords:** Bisphosphonates, γδ T cells, Liposomes, Immunotherapy, Sensitiser

## Abstract

Nitrogen-containing bisphosphonates (N-BP), including zoledronic acid (ZOL) and alendronate (ALD), have been proposed as sensitisers in γδ T cell immunotherapy in pre-clinical and clinical studies. Therapeutic efficacy of N-BPs is hampered by their rapid renal excretion and high affinity for bone. Liposomal formulations of N-BP have been proposed to improve accumulation in solid tumours. Liposomal ALD (L-ALD) has been suggested as a suitable alternative to liposomal ZOL (L-ZOL), due to unexpected mice death experienced in pre-clinical studies with the latter. Only one study so far has proven the therapeutic efficacy of L-ALD, in combination with γδ T cell immunotherapy, after intraperitoneal administration of γδ T cell resulting in delayed growth of ovarian cancer in mice. This study aims to assess the *in vitro* efficacy of L-ALD, in combination with γδ T cell immunotherapy, in a range of cancerous cell lines, using L-ZOL as a comparator. The therapeutic efficacy was tested in a pseudo-metastatic lung mouse model, following intravenous injection of γδ T cell, L-ALD or the combination. *In vivo* biocompatibility and organ biodistribution studies of L-N-BPs were undertaken simultaneously. Higher concentrations of L-ALD (40–60 μM) than L-ZOL (3–10 μM) were required to produce a comparative reduction in cell viability *in vitro,* when used in combination with γδ T cells. Significant inhibition of tumour growth was observed after treatment with both L-ALD and γδ T cells in pseudo-metastatic lung melanoma tumour-bearing mice after tail vein injection of both treatments, suggesting that therapeutically relevant concentrations of L-ALD and γδ T cell could be achieved in the tumour sites, resulting in significant delay in tumour growth.

## Graphical abstract

**Figure:**
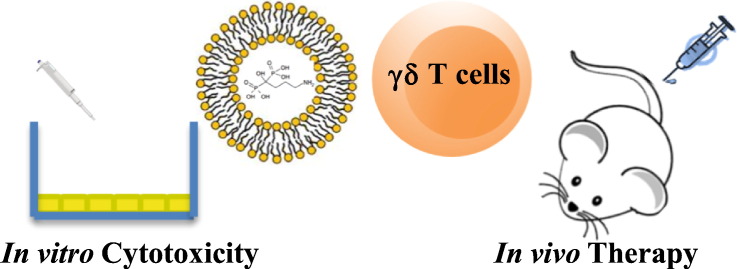

## 1 Introduction

Circulating gamma delta (γδ) T cells represent 1–10% of all peripheral blood T lymphocytes [1] and predominantly express the Vγ9Vδ2 T cell receptor (TCR) [2]. They recognize non-peptide phosphoantigens (PAgs) such as isopentyl pyrophosphate (IPP) [3]. In human cells, PAgs are generated *via* the mevalonate pathway, which is generally upregulated in transformed cells [4]. Vγ9Vδ2 T cells play an important role in cancer immunosurveillance [5] and have been used clinically in adoptive immunotherapy of cancer [6], [7], [8], [9], [10], [11]. Sensitisation approaches in immunotherapy have been sought to improve therapeutic outcomes. Nitrogen-containing bisphosphonates (N-BPs), such as zoledronic acid (ZOL) or alendronate (ALD), are known to inhibit farnesyl pyrophosphate (FPP) synthase, an enzyme in the mevalonate pathway, in cancer cells, causing intracellular accumulation of PAgs [12]. Exposure of Vγ9Vδ2 T cells to PAgs results in their activation *via* release of pre-formed perforin, granzymes and cytokines, and can lead to direct elimination of tumour cells [13].

It has been shown that pre-treatment of tumour cells with low concentrations of N-BPs, can sensitise them to killing by Vγ9Vδ2 T cells, resulting in an overall additive or synergistic cytotoxicity *in vitro* [14], [15], [16], [17], [18], *in vivo* [19], [20], [21], [22], [23], [24], [25], [26] and in clinical studies [8], [9], [27]. Therapeutic efficacy of N-BPs is hampered by their rapid renal excretion and high affinity for bone [28]. Improved pharmacokinetic profile and enhanced passive accumulation and retention within solid tumours has been achieved by encapsulation of ZOL and ALD into liposomes (L-ZOL and L-ALD) [29], [30]. L-ZOL was able to sensitise a number of ovarian cancer cell lines to destruction by Vγ9Vδ2 T cells *in vitro* [23]. However, its use *in vivo* was prohibited by the profound toxicity and sudden mice death [23], [29]. Several studies have reported the use of L-ALD for therapeutic applications in cancer [31] and inflammatory conditions [32], [33], [34], [35] pre-clinically. L-ALD has been shown to be effective when used with Vγ9Vδ2 T cells in an ovarian cancer model *in vivo* [23]. A clinical study is due to commence to evaluate the use of L-ALD in preventing coronary artery restenosis [36].

While several studies have reported the use of L-ALD or L-ZOL as a monotherapy in cancer models [31], [37], [38], [39], only one previous study has explored L-ALD in combination with γδ T cells in tumour mouse model, following local (IP) administration of γδ T cells to treat ovarian tumours [23]. In this study we hypothesise that systemic administration of γδ T cells is able to result in significant tumour growth delay when combined with L-ALD therapy in a pseudo-metastatic lung melanoma model. Additionally, the *in vivo* toxicity and biodistribution of L-ZOL and L-ALD has not been directly compared before. The aim of this study is to evaluate the *in vitro* potency, *in vitro* and *in vivo* efficacy of liposomal alendronate in combination with γδ T cell immunotherapy in cancerous cell lines and mice, respectively. In addition to efficacy studies, whole body organ biodistribution and *in vivo* toxicity were performed, bringing this formulation a step further towards biopharmaceutical development and evaluation in pre-clinical models.

## 2 Materials and methods

### 2.1 Materials

1,2-distearoyl-sn-glycero-3-phosphocholine (DSPC) and 1,2-dipalmitoyl 1,2-distearoyl-sn-glycero-3-phosphoethanolamine-*N*-[methoxy(polyethylene glycol)-2000] (ammonium salt) (DSPE-PEG2000) were obtained from Lipoid (Germany). 1,2-distearoyl-*sn*-glycero-3-phosphoethanolamine-*N*-diethylenetriaminepentaacetic acid (ammonium salt) (DSPE-DTPA) was purchased from Avanti Polar Lipids, Inc. (USA). Dextrose, cholesterol, sodium chloride, phosphate buffered saline (PBS) tablet, *N*-(2-Hydroxyethyl)piperazine-*N*′-(2-ethanesulfonic acid) (HEPES), methanol (analytical reagent grade), chloroform (analytical reagent grade), isopentane (analytical reagent grade), diethyl ether (analytical reagent grade) and Sephadex G75 were purchased from Sigma (UK). Zoledronic acid was a kind gift from Novartis (Switzerland). PD-10 desalting column was obtained from GE Healthcare Life Sciences (UK). Snake Skin® dialysis tubing (MWCO 10000 Da) was purchased from Thermo-fisher (USA). Dulbecco's modified Eagle's medium (DMEM), Glutamax™ and antibiotic-antimycotic solution were purchased from Invitrogen (UK). Foetal Bovine Serum (FBS) was purchased from First Link (UK). Thiazolyl blue tetrazolium bromide (MTT) and alendronate sodium trihydrate, were obtained from Alfa Aesar (UK). DMSO was obtained from Fisher (UK). Human IFN-γ ELISA Ready-set-go kit was purchased from eBiosciences (UK). Mouse TNF (Mono/Mono) ELISA set was purchased from BD Biosciences (USA). Indium-111 chloride was obtained from Mallinckrodt (NL). Thin layer chromatography (TLC) strips for radio-labelling were purchased from Agilent Technologies UK Ltd. (UK). Isoflurane (IsoFlo®) for anaesthesia was purchased from Abbott Laboratories Ltd. (UK). All reagents were used without further purification.

### 2.2 Preparation of liposomes

Liposomes were prepared using the thin film hydration (TFH) method. DSPC, cholesterol and DSPE-PEG2000 (55:40:5 mole ratio) were added to a 25 ml round-bottom flask and 2 ml chloroform/methanol (4:1 v/v) was added. A thin lipid film was formed upon removal of the solvent under reduced pressure using a rotary evaporator (Rotavapor® R-210, Buchi UK). The lipid film was flushed with nitrogen to remove any remaining traces of organic solvent. The lipid film was then hydrated with 1 ml of PBS, adjusted to pH 7.4. The liposome suspension was left for 1 h at 60 °C and was vortexed (Vortex genie 2, Scientific Industries Inc., USA) every 15 min [40]. The resulting suspension was stored at 4 °C. The size and polydispersity (PDI) of the liposomes were reduced with serial extrusion steps. The liposome suspension was extruded using the mini-extruder (Avanti Polar Lipids, USA) through polycarbonate membranes (Avanti Polar Lipids, USA) with pore sizes 0.8 μm (5 ×), 0.2 μm (5 ×), 0.1 μm (10 ×) and 0.08 μm (15 ×), above the phase transition temperature for the lipid. When formulating L-ZOL and L-ALD, the lipid films were hydrated with either 100 mM ZOL or 100 mM ALD in HEPES Buffered Saline (HBS; 20 mM HEPES and 150 mM NaCl, pH 7.4), and free ZOL and ALD was removed by dialysis against HBS using a 10,000 Da MWCO dialysis bag. Liposomes were prepared at a final lipid concentration of 25 mM.

### 2.3 Cancer cell line culture conditions

The cell lines PANC-1 (CRL-1469™), PANC0403 (CRL-2555™) were obtained from ATCC®. A375Ppuro and A375Pβ6 cell lines were created using the human melanoma cell line A375P (CRL-3224™), which was infected with pBabe retroviruses encoding puromycin resistance alone (A375Ppuro) or in combination with cDNA for human β6 integrin (A375Pβ6), as previously reported [41]. The A375Ppuro and A375Pβ6 were a kind gift from Dr. John Marshall (QMUL). All cell lines were maintained at 37 °C, 5% CO_2_ and 5% relative humidity. Advanced RPMI or DMEM media were used, both of these were supplemented with 10% FBS, 1% Glutamax and 1% Penicillin/Streptomycin.

### 2.4 Treatment of cancer cell lines with N-BPs in monotherapy studies

The cell lines A375Ppuro and PANC-1 were seeded in a 96-well plate at a seeding density of 10,000 cells/well. Cells were treated with 0.01–100 μM ZOL or ALD, 20–200 μM empty liposomes (EL) or were left untreated. After 24, 48 or 72 h incubation, the cell viability was assessed with MTT, as described below.

### 2.5 Treatment of cancer cell lines with N-BPs/liposomal N-BPs and γδ T cells in combination therapy studies

The cell lines A375Ppuro, A375Pβ6, PANC-1 and PANC0403 were seeded at 50,000 cells/well in a 96-well plate. Confluent monolayers of each cancer cell line were treated for 24 h with ZOL, ALD, L-ZOL or L-ALD at concentrations of 3–10 μM (ZOL and L-ZOL), 40–60 μM (ALD and L-ALD) or were left untreated. With regards to L-ZOL and L-ALD, the concentrations used indicate the amount of encapsulated ZOL and ALD after purification. As a control, cells were also treated with EL at concentrations of 36.5–219 μM After 24 h, the treatments were removed and the monolayers were then co-cultured with 2.5 × 10^5^ *ex vivo* expanded γδ T cells (or γδ T cell culture media as a control) per well for a further 24 h. Cell viability was assessed with MTT as described below.

### 2.6 MTT assay

MTT (3-(4,5-dimethylthiazol-2-yl)-2,5-diphenyltetrazolium bromide) solution was prepared in PBS at a concentration of 5 mg/ml and was diluted in media (1:6 *v*/v) prior to use. The supernatant of each well was removed and MTT solution (120 μl) was added to each well. The plates were then incubated at 37 °C and 5% relative humidity for 3 h. The MTT solution from each well was removed and DMSO was added to solubilise (200 μl/well for 96 well) the crystals formed and this was incubated for 5 min at 37 °C, to eliminate entrapped air bubbles. The absorbance was read at 570 nm with subtraction readings at 630 nm to normalise for cell debris (FLUOStar Omega, BMG Lab Tech). Percentage cell viability (%) was calculated as a percentage of untreated cells (equation in SI). Cell viability was expressed as mean ± standard deviation (*n* = 5).

### 2.7 Determination of IFN-γ concentration with ELISA

Supernatant from the co-culture assay was removed from each of the wells immediately before the cytotoxicity assay was performed. The supernatant was centrifuged to remove the γδ T cells and was stored at − 80 °C until required. Supernatants were diluted 1:40 and analysed using a human IFN-γ ELISA Ready-set-go-kit as per the manufacturer's protocol.

### 2.8 Radiolabelling of liposomes

DSPC: cholesterol: DSPE-PEG_2000_:DSPE-DTPA (54:40:5:1 mole ratio) liposomes were prepared with the TFH method, as described above, then radiolabelled with ^111^In [42]. Briefly, the required volume of ^111^In, containing 1 MBq or 10–15 MBq per mouse for gamma counting or SPECT/CT imaging studies, respectively, was added to 2 M ammonium acetate buffer (one-ninth of the reaction volume, pH 5.5). This was then added to the liposome sample (100 μl of 20 mM liposomes/mouse) to give a final ammonium acetate concentration of 0.2 M. The mixture was incubated for 30 min at room temperature with vortexing every 10 min. The reaction was quenched by the addition of 0.1 M EDTA solution to the mixture (5% v/v of the reaction mixture) to chelate free ^111^In. Unbound ^111^In:EDTA was removed using NAP-5 desalting columns equilibrated with PBS with the liposomes collected in fractions 1–3 (~ 150 μl per injection dose).

### 2.9 Efficiency and stability of the radiolabelling in serum

Samples of the radiolabelled liposomes or ^111^In:EDTA were spotted in glass microfibre chromatography paper impregnated with silica gel. These strips were then developed using a mobile phase of 50 mM EDTA in 0.1 M ammonium acetate. Strips were placed on a multi-purpose storage phosphor screen (Cyclone ®, Packard, Japan) and kept in an autoradiography cassette (Kodak Biomax Cassette ®) for 10 min. Quantitative autoradiography counting was then carried out using a cyclone phosphor detector (Packard ®, Australia). The labelling stability was tested by incubation of the radio-conjugates in the presence or absence of foetal bovine serum (FBS). Samples were diluted in 50% FBS or PBS [1:2 (v/v)], and incubated for 24 h at 37 °C. The percentage of ^111^In (immobile spot) still conjugated to the liposomes was evaluated by TLC, using the same protocol, as described above.

### 2.10 Animal models

All animal experiments were performed under the authority of project and personal licences granted by the UK Home Office and the UKCCCR Guidelines (1998). Male NOD SCID gamma (NSG) mice (~ 20 g), 4–6 weeks old, were obtained from Charles River (UK). Subcutaneous (s.c.) tumours were established by injecting 5 × 10^6^ cells A375Pβ6 in 100 μl PBS into each of the rear-flanks of the mouse. The size of the tumour was measured using callipers and tumour area and volume could then be determined using the equation Tumour volume = width*width*length*(3.14/6). Experiments were performed when tumours reached ~ 300 mm^3^.

### 2.11 *In vivo* toxicity studies of L-ALD and L-ZOL in NSG mice after a single injection

Non-tumour bearing NSG mice were intravenously injected with 0.1 μmol L-ZOL or 0.5 μmol L-ALD. After 72 h, the mice were sacrificed and the toxicity of L-ZOL and L-ALD assessed using the methods below.

#### 2.11.1 Spleen weight

The spleens were excised from each mouse and weighed using a laboratory balance (GeniusME, Sartorius, Germany).

#### 2.11.2 Haematological profile

Whole blood samples were obtained *via* cardiopuncture using K_2_EDTA as an anti-coagulant. Fresh blood smears were made using 5 μl blood and the haematological profiles of these samples were performed by the Royal Veterinary College (London, UK).

#### 2.11.3 Serum biochemistry

Serum was obtained from some of whole blood samples by allowing the blood to clot and centrifuging at for 15 min at 1500 g. The serum biochemistry profiles were performed by the Royal Veterinary College (London, UK).

#### 2.11.4 TNF-α serum levels

TNF-α ELISA was performed on serum samples (diluted 1:3) using a mouse TNF-α (Mono/Mono) ELISA set as per the manufacturer's protocol.

#### 2.11.5 Organ histology

Organs were immediately fixed in 10% neutral buffer formalin as 5 mm^2^ pieces. These pieces were then paraffin-embedded and sectioned for haematoxylin and eosin stains (H&E) according to standard histological protocols at the Royal Veterinary College. The stained sections were analysed with a Leica DM 1000 LED Microscope (Leica Microsystems, UK) coupled with CDD digital camera (Qimaging, UK).

#### 2.11.6 Survival

Mice were injected with 0.1 μmol L-ZOL (*n* = 2) or 0.5 μmol L-ALD (*n* = 10) and observed for weight loss and overall appearance daily.

### 2.12 *In vivo* toxicity of L-ALD in NSG mice after multiple injections

Non-tumour bearing NSG mice were injected with at one week intervals with 0.5 μmol L-ALD for a total of three doses. The blood, serum and organs of the mice were analysed as above, with the mice sacrificed 72 h after the final injection.

### 2.13 Whole body SPECT/CT imaging of radiolabelled liposomes in A375Pβ6-tumour bearing mice

Each mouse was injected with radiolabelled liposomes at 2 μmol each, containing 1 MBq or 10–15 MBq, for biodistribution and SPECT/CT studies, respectively, *via* tail vein injection. Mice were imaged with nanoSPECT/CT scanner (Bioscan ®, USA) at different time points; immediately after the i.v. administration (0–30 min), 4 h and 24 h. For each mouse, a tomography was initially done (45 Kvp; 1000 ms) to obtain parameters required for the SPECT and CT scanner, including the starting line, finish line and axis of rotation of the acquisition. SPECT scans were obtained using a 4-head scanner with 1.4 mm pinhole collimators using the following settings: number of projections: 24; time per projection: 60 s and duration of the scan 60 min. CT scans were obtained at the end of each SPECT acquisition using 45 Kvp. All data were reconstructed with MEDISO (medical Imaging System) and the combining of the SPECT and CT acquisitions were performed using PMOD® software.

### 2.14 Gamma scintigraphy of radiolabelled liposomes in A375Pβ6-tumour bearing mice

After 24 h, mice were sacrificed and the major organs (brain, lung, liver, spleen, kidney, heart, stomach and intestine), muscle, skin, bone (femur), carcass and tumours were collected, weighed and placed in scintillation vials. Additionally, 5 μl blood samples were collected at various time points (5, 10, 30, 60, 240 and 1440 min). Each sample was analysed for [^111^In] specific activity using an automated gamma counter (LKB Wallac 1282 Compugamma, PerkinElmer, UK) together with dilutions of injected dose with dead time limit below 60%. The gamma rays emitted by the radioisotope were detected, quantified and corrected for physical radioisotope decay by the gamma counter. Radioactivity readings (counts per minute- CPM) were plotted as percentage of injected dose per organ (%ID/organ) or percentage of injected dose per gram of tissue (%ID/g). The data were expressed as the mean of triplicate samples ± SD.

### 2.15 Therapy study

Male NSG mice (4–6 weeks) were inoculated with 5 × 10^6^ A375Pβ6.luc by i.v injection to form experimental metastatic lung tumours. Bioluminescence imaging of mice was carried out on Day 6 as described above and mice were divided into 4 treatment groups: naïve, L-ALD, γδ T cells and L-ALD and γδ T cells combination treatment. Doses used in therapy experiments were 0.5 μmol of ALD/mouse (L-ALD) and 1 × 10^7^ cells/mouse (γδ T cells), all injected *via* the tail vein. Three doses of each treatment were given at one week intervals on days 7, 14 and 21. In the case of the combination treatment, mice were pre-injected with L-ALD (days 6, 13, and 20) then injected with γδ T cells (days 7, 14, and 21). Tumour growth was monitored by bioluminescence imaging twice weekly (days 6, 10, 13, 17, 20, 24, 27), as described above.

### 2.16 Determination of IFN-γ concentration with ELISA

Animals from the therapy study were sacrificed and sera was analysed for human IFN-γ. Sera were diluted 1:2 and analysed using a human IFN-γ ELISA Ready-set-go-kit as per the manufacturer's protocol.

### 2.17 Statistics

For all experiments, data were presented as mean ± SD, where n denotes the number of repeats. Independent variable Student *t*-tests were performed using IBM SPSS version 20 for *in vitro* cytotoxicity studies. For *in vivo* studies, significant differences were examined using one-way ANOVA. The t-value, degrees of freedom and two-tailed significance (*p*-value) were determined. *p < 0.05, **p < 0.01 and ***p < 0.001.

## 3 Results

### 3.1 L-ZOL and L-ALD of comparable size and drug loading were prepared

L-ZOL and L-ALD composed of DSPC:cholesterol:DSPE-PEG_2000_ (55:40:2 mole ratio) were formulated using the Thin Film Hydration (TFH) method (Fig. S1), extruded and purified using dialysis. Liposomes were also prepared using the reverse phase evaporation (RVE) method as a comparison, but as liposomes produced by the two methods had the same characteristics, TFH method was used to produce liposomes for all subsequent experiments. Liposomes exhibited a hydrodynamic size of 155.4–159.0 nm, with narrow polydispersity index (PDI of 0.045–0.104) and slightly negative zeta potential (− 11.7 to − 14.0 mV) (Table S1). Methods were developed to quantify the amount of ZOL and ALD encapsulated into liposomes, as described in the supplementary information, using UV–Vis or HPLC (for ZOL quantification) (Fig. S2), copper sulphate-based UV spectroscopy method or o-phthalaldehyde (OPA)-based fluorescence methods (for ALD quantification) (Fig. S3). Our results showed that both ZOL and ALD had similar encapsulation efficiencies (% EE) ranging from 5.2–6.4% (Table S2). Drug loading of 0.23–0.27 mmol ZOL or ALD per mmol lipid was obtained (*p* > 0.05).

### 3.2 *In vitro* anti-tumour activity of ZOL is more potent than ALD

**Fig. 1:**
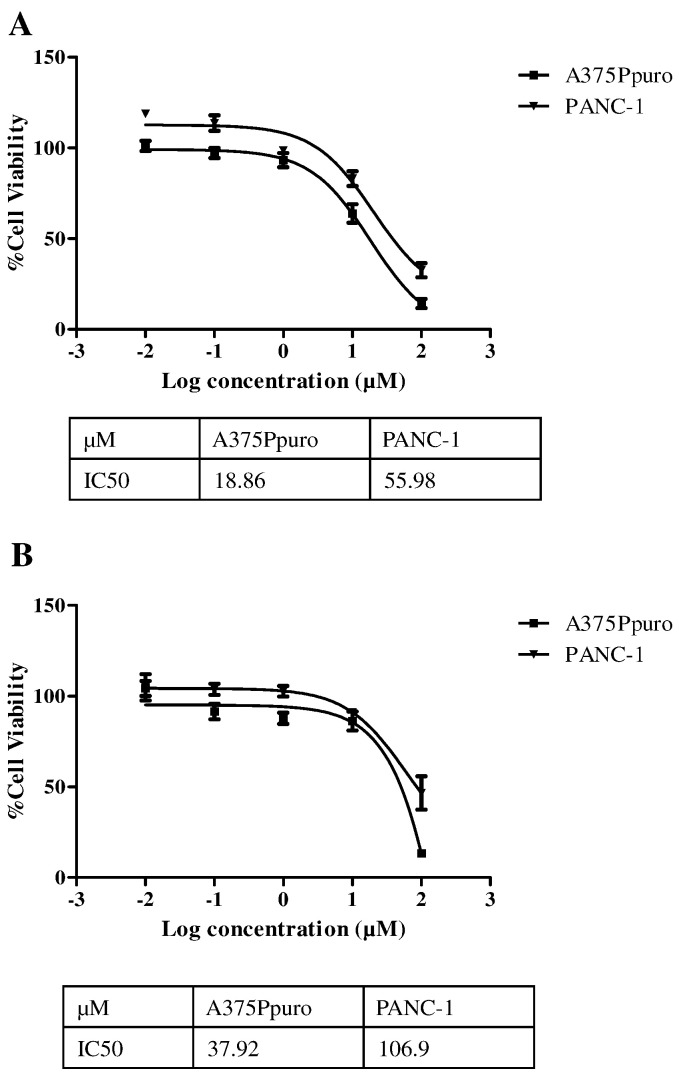
IC50 values of N-BPs after 72 h incubation for different human cancer cell lines. The IC_50_ values were determined for the melanoma cancer cell line A375Ppuro and the pancreatic cancer cell line PANC-1 incubated with **(A)** ZOL or (B) ALD for 72 h. IC 50 are in the order of PANC-1 > A375Ppuro for both ALD and ZOL. Higher IC50 values were obtained for ALD than ZOL. R^2^ values of 0.9988 (ALD PANC-1 and ZOL A375Ppuro). 0.9736 (ALD A375Ppuro) and 0.9718 (ZOL PANC-1) were obtained. Data was expressed as mean ± SD (*n* = 5).

The cytotoxicity of ZOL and ALD as a monotherapy was assessed using the melanoma cell line A375Ppuro and the pancreatic cell line PANC-1. Additionally empty liposomes (EL) were tested as controls. These results would enable non-toxic ranges of both N-BPs and EL to be established for use in co-culture studies with γδ T cells. ZOL and ALD were tested in the range of 0.01–100 μM for 24, 48 and 72 h of incubation. Time- and dose-dependent cytotoxicity was elicited by both N-BPs. Cell viability results for 24, 48 and 72 h are shown in Figs. S5 and S6. In order to compare the cytotoxicity of the two N-BPs, cell viability at 72 h was used to calculate the IC_50_ values for ZOL and ALD in the two cell lines (Fig. 1). IC_50_ values of ZOL were 18.86 μM and 55.98 μM for A375Ppuro and PANC-1, respectively. IC_50_ values of ALD were 37.92 μM and 106.9 μM for same cell lines. It was concluded that PANC-1 cells were more resistant than A375Ppuro to the direct cytotoxic action of N-BPs. Moreover, ZOL was more potent that ALD in both cell lines. ZOL and ALD concentration ranges selected for co-culture studies with γδ T cells were 3–10 μM and 40–60 μM, respectively. It is worth mentioning that such high IC_50_ values for both drugs suggests that neither is suitable for use as an anti-cancer agent for non-osseous tumours.

In case of EL, a reduction in cell viability of PANC-1 was observed at concentrations > 200 μM with cell viabilities of 87.0 ± 1.5% and 87.9 ± 8.8% after 48 and 72 h, respectively (Fig. S7). A375Ppuro cells proved more sensitive to non-specific toxic effects of EL with cell viabilities of 72.5 ± 5.7% and 45.5 ± 3.4% under similar treatment conditions (Fig. S7). In co-culture studies with γδ T cells, cancer cells will be treated with liposomal formulations for only 24 h at concentrations < 40 μM (ZOL) and < 240 μM (ALD), after which the drug is removed. Cells will be further incubated for 24 h with γδ T cells. This incubation protocol is unlikely to result in significant non-specific toxicity from the carrier itself.

### 3.3 L-ZOL and L-ALD can sensitise cancer cells to destruction by γδ T cells in co-culture studies

Free and liposomal N-BPs were then used in combination with γδ T cells, to assess whether pre-treating the cells with the N-BPs would sensitise cancer cells for destruction by γδ T cells. The isolation and expansion protocol used to generate and characterise Vγ9Vδ2 T cells is described in supplementary information (Figs. S8 and S9). In this experiment, two melanoma (A375Ppuro and A375Pβ6) and two pancreatic (PANC-1 and PANC0403) cancer cell lines were used. Each pair of cells included an αvβ6 integrin positive and negative cell line to allow for future use of targeted liposomes. We hypothesise that free and liposomal N-BPs or γδ T cells are not toxic to cancer cells when used individually under the conditions tested, but their pre-treatment with N-BPs sensitise them to killing by γδ T cells. A scheme of the treatment protocol is shown in Fig. S10.

**Fig. 2:**
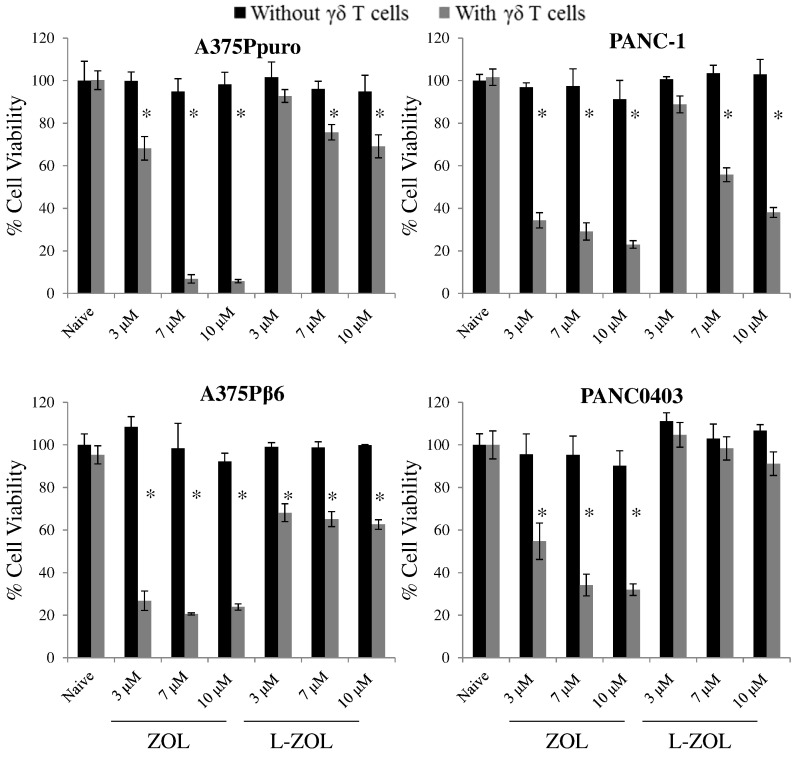
Viability of human cancer cell lines after incubation with γδ T cells and L-ZOL. Cells were treated with ZOL or L-ZOL for 24 h at concentrations between 3 and 10 μM. The treatments were then removed and replaced with 2 × 10^5^ γδ T cells for an additional 24 h, before an MTT assay was performed to determine residual tumour cell viability. The ZOL and L-ZOL were used at non-toxic concentrations, in the absence of γδ T cells. No background toxicity was found for γδ T cells without N-BP. However, a dose dependent toxicity was found in cells pre-treated with ZOL or L-ZOL (ZOL > L-ZOL), except for PANC0403. Data was expressed as mean ± SD (*n* = 5). *p < 0.05, (Student's *t-*test *vs.* naive).

Initial experiments focused on ZOL and L-ZOL. As expected, ZOL, L-ZOL or γδ T cells exerted no cytotoxic effect against these tumour cells when used individually, at previously determined sub-toxic concentrations (black bars, Fig. 2). By contrast, a significant and dose-dependent reduction in cell viability was seen when free ZOL or L-ZOL was used to sensitise tumour cells to subsequent addition of Vγ9Vδ2 T-cells. This toxic effect was more evident with free ZOL than with L-ZOL (grey bars, Fig. 2). In keeping with this, PANC0403 cells appeared to be resistant to L-ZOL/γδ T cells combination therapy, whereas free ZOL could effectively sensitise these tumour cells.

**Fig. 3:**
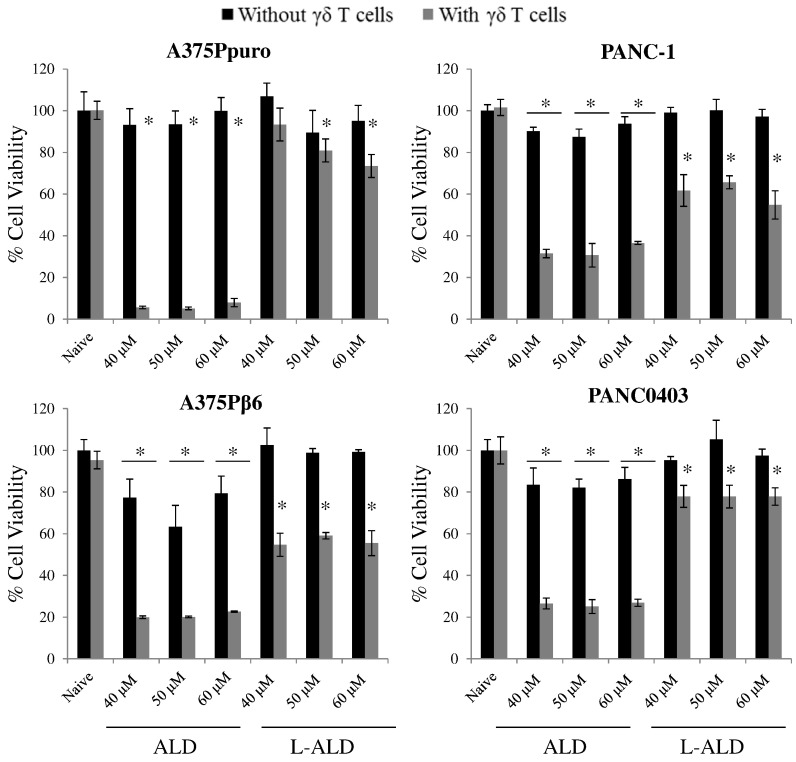
Cell viability of human cancer cell lines after incubation with γδ T cells and L-ALD. Cells were treated with ALD or L-ALD for 24 h at concentrations between 40 and 60 μM. These agents were then removed and replaced with 2 × 10^5^ γδ T cells for an additional 24 h, before an MTT assay was performed to measure residual tumour cell viability. The ALD and L-ALD were used at non-toxic concentrations, in the absence of γδ T cells. No background toxicity was found for γδ T cells without N-BP. However, a non-dose dependent toxicity, in the range tested, was found in cells pre-treated with ALD or L-ALD (ALD > L-ALD). Data was expressed as mean ± SD (*n* = 5). *p < 0.05, (Student's *t-*test *vs.* naive).

Next, we evaluated the ability of ALD or L-ALD to sensitise tumour cells to Vγ9Vδ2 T-cells. In agreement with cell viability studies using N-BPs as monotherapy, higher concentrations of ALD and L-ALD than for ZOL, were required to induce reductions in cancer cell viability in the co-cultures. Cell viabilities of 5–37% and 55–93% were obtained for ALD and L-ALD, respectively, when used with γδ T cells treatment, at 40–60 μM ALD (grey bars, Fig. 3).

Unlike ZOL and L-ZOL, no dose-dependency was observed in the case of ALD or L-ALD treatment, possibly due to the narrower range used than in the ZOL study. A slight but significant reduction in cell viability was observed when cells were treated with free ALD, in some of the conditions, despite the absence of γδ T cells (~ 60–90% % cell viability, *p* < 0.05). This is presumably due to the high ALD concentrations used compared to ZOL. No reduction in cell viability was found when cells were pre-treated with EL and then γδ T cells, at equivalent concentrations used with the drug (Fig. S11). These studies further confirmed that the reduction in cancer cell viability is specific to N-BPs ability to sensitise cells to γδ T cells. It was also concluded that free N-BPs can sensitive cancer cells more efficiently than their liposomal formulations. This is not surprising as encapsulation of the drug within liposomes is likely to slow down its release (Fig. S4), causing a delayed onset of action *in vitro* as has been reported for other drugs [43].

### 3.4 Interferon (IFN)-γ production is increased proportionally to cell kill

**Fig. 4:**
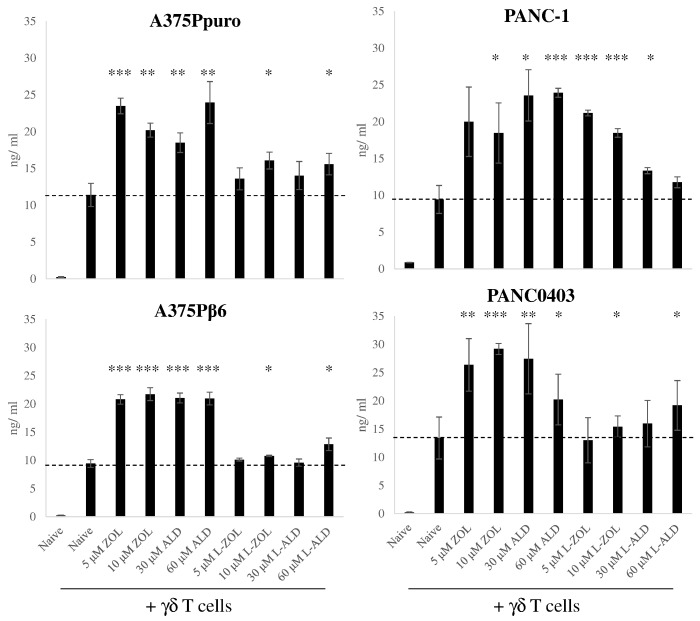
IFN-γ production by γδ T cells after incubation with cancer cells. IFN-γ ELISA was performed on supernatant removed from the co-culture experiment, prior to the MTT assay, for each of the cancer cell lines. The quantity of IFN-γ produced by the γδ T cells for each of the different pre-treatment conditions is expressed as ng/ml. Free ZOL or ALD led to an increased production of IFN-γ compared to γδ T cells incubated with untreated cells. Pretreament with L-ZOL or L-ALD led to a smaller or no increase in IFN-γ production. Data was expressed as mean ± SD (*n* = 3). *p < 0.05, **p < 0.01, ***p < 0.001. (Student's *t-*test *vs.* γδ T cells alone).

The IFN-γ concentrations in the co-culture supernatants were measured in order to further confirm that cell kill was specific to γδ T cell activation. In all cases, the use of free N-BPs with γδ T cells led to a significant increase in IFN-γ levels compared to γδ T cells treatment alone (18–27 *vs.* ~ 10 ng/ml) (Fig. 4). Agreeing with the cell viability results, IFN-γ levels were approximately two-fold higher in cells pre-treated with free ZOL or ALD than their liposomal formulations (Fig. 4). Only some L-ZOL or L-ALD treatment groups showed significant increases in IFN-γ levels, but in a random manner. The good correlation between cell viability and IFN-γ concentration suggests that cell kill is due to the activation of γδ T cells.

### 3.5 *In vivo* toxicity of L-ZOL and L-ALD following a single injection in NOD scid gamma (NSG) mice

It has been reported by Shmeeda et al., that L-ZOL resulted in sudden death of mice (BALB/c and Sabra) when used *in vivo* [29]. The use of L-ZOL and L-ALD in NSG mice has not been reported. NSG mice have been increasingly used for *in vivo* studies and may have different profiles to other mouse strains, as they are more immunocompromised. In the work, we used immunocompromised mice, as the aim is to be able to perform therapy study against human cancers in combination with human γδ T cells, using these mice. Immuno-competent mice could not be used to grow human tumours or to inject γδ T cells, hence were not used. In this study, a direct comparison was conducted for L-ZOL and L-ALD using the parameters outlined in Shmeeda et al., following a single injection. Based on IC_50_ values obtained *in vitro*, a 5-fold higher dose of L-ALD (0.5 μmol ALD/mouse) than L-ZOL (0.1 μmol ZOL/mouse) was used *in vivo*. Mice were sacrificed 72 h post single i.v. injection of liposomal N-BPs (L-N-BPs: L-ZOL or L-ALD), since in previous studies mice death was observed at 5 days L-ZOL post-injection. Parameters monitored and findings obtained are detailed below.

#### 3.5.1 Injection of L-ZOL or L-ALD leads to splenomegaly

**Fig. 5:**
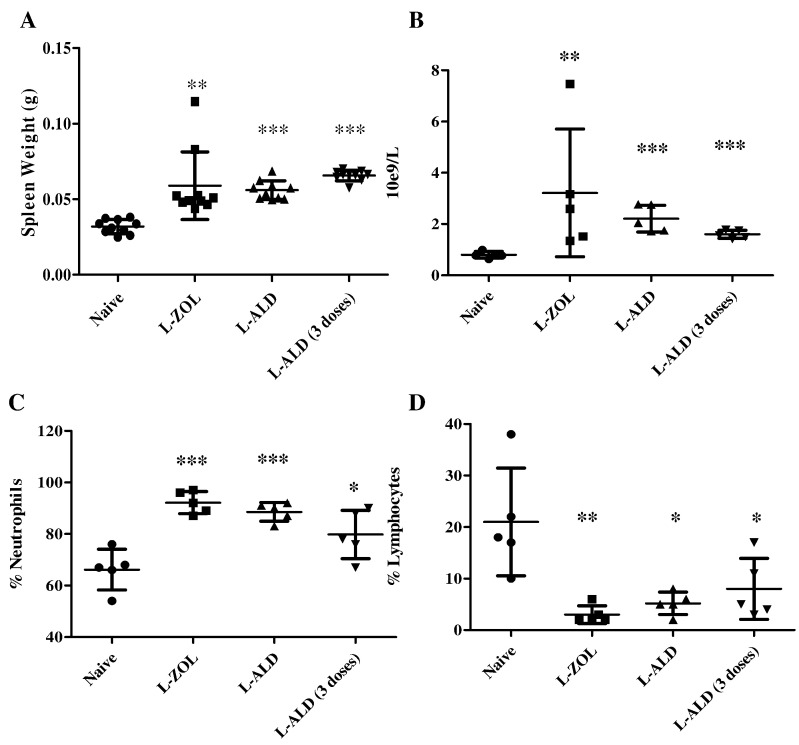
Effects of L-ZOL and L-ALD on blood counts and spleen. NSG mice were injected with 0.1 μmol L-ZOL or 0.5 μmol L-ALD. After 72 h, the mice were sacrificed. (A) The spleen was removed and weighed. A significant increase in spleen weight could be seen in the case of both L-ZOL and L-ALD. Blood counts were performed by automatic counting. An increase in (B) WBC and (C) % Neutrophils was seen when injected with L-ZOL or L-ALD, while a decrease in (D) % Lymphocytes was observed. (Data were expressed as mean ± SD (*n* = 10 and *n* = 5 for spleen weight and blood counts, respectively) *p < 0.05, **p < 0.01, ***p < 0.001. (Student's *t-*test *vs.* naive).

Spleens of mice injected with L-ZOL weighed significantly more (0.06 ± 0.02 g) than those of control mice (0.03 ± 0.004 g) (*p* < 0.01) (Fig. 5A). Additionally, the spleens of mice injected with L-ALD also displayed significant splenomegaly *vs.* control spleens (0.06 ± 0.01 g, *p* < 0.001).

#### 3.5.2 Haematological analysis

It has been suggested that the systemic toxicity of L-ZOL in mice is haematologically related [29]. Changes in the full blood count profiles have previously been reported for L-ZOL. Additionally, liposomal N-BPs are known to have macrophage depleting effects. Agreeing with the previously reported results, L-ZOL caused leucocytosis, neutrophilia and lymphocytopenia (Fig. 5B–D). White blood cells count and % neutrophils increased from 0.77 ± 0.15 × 10^9^/L and 66.2 ± 7.9% in control mice to 3.22 ± 2.49 × 10^9^/L (*p* < 0.01) and 92.2 ± 4.3% (*p* < 0.001), in L-ZOL group. Matching profiles for L-ZOL and L-ALD were obtained. The complete haematological profile for L-ZOL and L-ALD is shown in Table 1.

#### 3.5.3 Serum biochemistry profile

The biochemistry profile of mice injected with L-ZOL or L-ALD was studied. Mild but non-significant hypocalcaemia and elevated Blood Urea Nitrogen (BUN) were previously reported for L-ZOL [29]. The complete serum biochemistry profiles are shown in Table S3. In our study, L-ZOL and L-ALD did not display any significant differences to each other or to control mice. L-ZOL (6.78 ± 0.69 mmol/L) however resulted in small but significant reduction in urea compared to the control (8.42 ± 0.97 mmol/L) (*p* < 0.05). Additionally, L-ALD (27.60 ± 1.67 g/L) led to a significant reduction in albumin levels compared to control mice (31.6 ± 1.67 g/L) (*p* < 0.01).

#### 3.5.4 TNF-α levels are not increased in mice treated with L-ZOL or L-ALD

It has been shown the L-ZOL can cause a moderate non-significant increase in TNF-α levels *in vivo* [29]. An ELISA was performed on the serum to determine TNF-α levels. Mice injected with L-ZOL and L-ALD did not result in detectable levels of TNF-α in serum. As a positive control, serum from LPS challenged mice were also tested and produced TNF-α levels of 1.6 ng/ml. This difference may be due to the mice been sacrificed at an earlier timepoint than in the reported study, or due to the different strain of mouse used.

#### 3.5.5 No histological abnormalities seen in mice post i.v. injection of L-ZOL or L-ALD

Histological examination of the major organs (heart, lung, liver, spleen and kidney) with H&E staining showed no obvious histological changes compared to control animals (Fig. S12), agreeing with the published study on L-ZOL [29].

#### 3.5.6 Mice treated with L-ZOL but not L-ALD experience sudden death 5 days post injection

Death of mice injected with L-ZOL (0.1 μmol ZOL), without warning sign, has been reported to occur 5–7 days after injection BALB/c and outbred Sabra mice [29]. Two NSG mice were injected in this study. Mice were found dead without showing signs of physical abnormalities or weight loss. It was judged unethical to inject more mice with this formulation. On the other hand, all mice injected with 0.5 μmol L-ALD (*n* = 10) showed 100% survival over the entire study duration (24 days).

### 3.6 Multiple and single dosing of L-ALD show comparable *in vivo* toxicity profiles in NSG mice

To mimic dosing regimen used in combination N-BP and γδ T cell immunotherapy studies, multiple dosing of L-ALD, with weekly intervals, was performed. The overall *in vivo* toxicity was compared to that of single administration. Mice were sacrificed 72 h after the final injection. The spleen weights (Fig. 5A), haematology (Fig. 5B–D and Table S4) and biochemistry (Table S5) profiles were not significantly different from values obtained with single L-ALD injection. This suggests that the toxicity from L-ALD was not cumulative.

### 3.7 *In vivo* whole body SPECT/CT imaging of EL

The effect that placing N-BPs into liposomes would have on their biodistribution was then studied. The cell line A375Pβ6 was chosen due to its favourable *in vivo* growth and greater ability to be sensitised to γδ T cells by L-ALD than the other cell lines screened. Liposomes were formulated to include 1% DSPE-DTPA and were labelled with ^111^In, which did not affect the physicochemical properties of the liposomes (data not shown). Initial labelling of 86.3% was achieved and in the presence of PBS or 50% FBS, 87.8 and 91.1% remained bound to the EL after 24 h, respectively (Fig. S13). Whole body SPECT/CT images of intravenously injected [^111^*In*]EL in A375Pβ6 subcutaneous tumour-bearing NSG mice were performed in order to track the biodistribution of EL over time. The mice were imaged at multiple time points up to 24 h post-injection as shown in Fig. S14. At early time-points, EL displayed high concentrations in the circulation, with activity located throughout the mouse at 0–30 min and to a slightly lesser extent at 4 h. At 24 h, accumulation of [^111^*In*]EL in liver and spleen was observed. The uptake in A375Pβ6 tumour could not be observed by this imaging modality, possibly due to prolonged blood circulation of the [^111^*In*]EL.

### 3.8 L-ZOL and L-ALD show similar tumour and organ biodistribution patterns *in vivo*

**Fig. 6:**
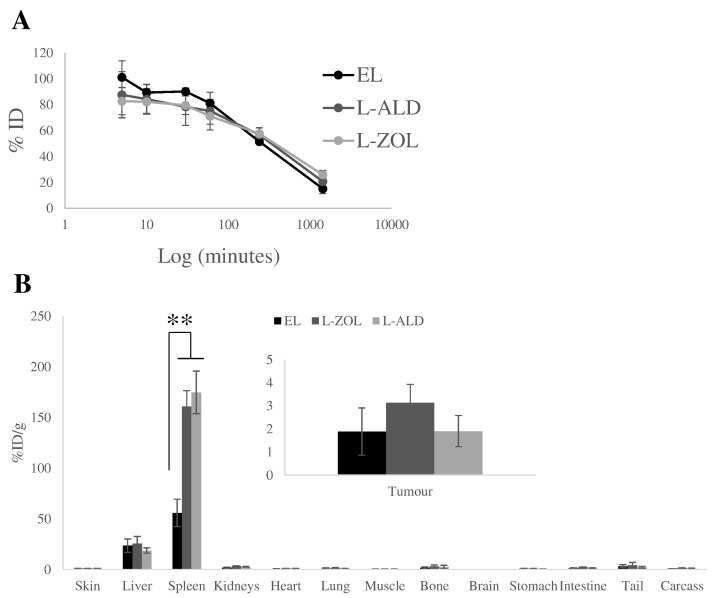
*In vivo* biodistribution of radiolabelled EL, L-ALD and L-ZOL in A375Pβ6 tumour bearing NSG mice after single dose administration via tail vein injection. NSG mice were inoculated bifocally with the A375Pβ6 cell line and were i.v. injected with ^111^In labelled liposomes at a dose of 2 μmol lipid/mouse. (A) Blood clearance profile of liposomes expressed as %ID. (B) Results were expressed as percentage injected dose per gram of organ (%ID/g organ) at 24 h after injection of 2 μmol liposome/mouse. L-ZOL and L-ALD was seen to have higher spleen accumulation than EL. Data are expressed as mean ± SD (*n* = 3) *p < 0.05, **p < 0.01. (Student's *t-*test *vs.* naive).

The organ biodistribution and tumour uptake profiles of [^111^*In*]EL, [^111^*In*]L-ZOL and [^111^*In*]L-ALD following i.v. injection were assessed quantitatively by gamma counting in A375Pβ6 subcutaneous tumour-bearing NSG mice. This was done in order to help understand the toxicity results obtained. Prolonged blood circulation profiles were not significantly different between the 3 formulations, with 71–81, 52–58 and 15–26%ID remaining in the blood at 1 h, 4 h and 24 h, respectively (Fig. 6A). Agreeing with SPECT/CT images, the liver and spleen were the organs with the highest liposome accumulation (Fig. 6B). Liver uptake was 23.5 ± 6.5, 25.4 ± 7.2 and 18.7 ± 2.7% ID/g for [^111^*In*]EL, [^111^*In*]L-ZOL and [^111^*In*]L-ALD, respectively, at 24 h. Spleen uptake was 55.8 ± 13.6, 144.1 ± 70.5 and 148.9 ± 61.1%ID/g for the same formulations. Both [^111^*In*]L-ZOL and [^111^*In*]L-ALD showed significantly 3-fold higher spleen uptake than [^111^*In*]EL, with no significant differences seen between [^111^*In*]L-ZOL and [^111^*In*]L-ALD. No significant differences in tumour uptake between the three formulations were found (~ 1.9–3.1% ID/g) (Fig. 6B, inset). The organ biodistribution profiles expressed as % ID/organ are displayed in Fig. S15. We believe that any differences in *in vivo* toxicity are not likely due to differences in pharmacokinetic profiles, since all liposomes exhibited similar size and surface charge.

### 3.9 Combinatory L-ALD and γδ T cell immunotherapy

**Fig. 7:**
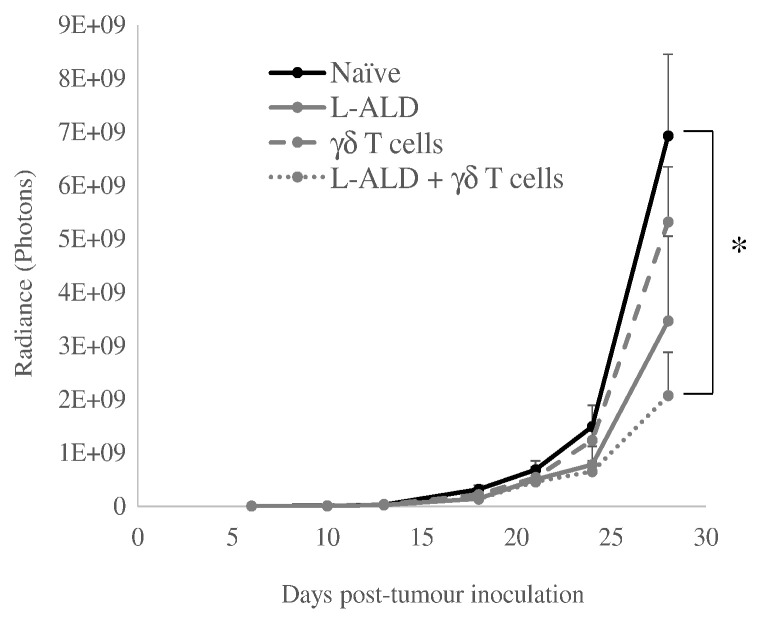
*In vivo* tumour therapy study. Pseudo-metastatic lung A375Pβ6 tumour bearing mice were treated with L-ALD (0.5 μmol ALD/mouse), 1 × 10^7^ γδ T cells/mouse or both, intravenously. Three treatments were given intravenously at one week intervals, commencing on day 6 post-tumour inoculation. Tumour progression was monitored by bioluminescence imaging. A significant reduction in tumour growth was observed for the combinatory immunotherapy. Data was expressed as mean ± SEM (*n* = 7). *p < 0.05, (Student's *t-*test *vs.* naïve).

To assess whether the dosing regimen of L-ALD used in the toxicity studies was sufficient to result in the potentiation of the immunotherapy, a tumour growth delay experiment was performed in the pseudo-metastatic lung tumour model, following i.v. administration of both therapeutic agents. At the start of treatment (day 6), all four groups (naïve, L-ALD, γδ T cells and L-ALD + γδ T cells) had the same average tumour size (3.4 × 10^6^ photons). After three treatments at one week intervals (day 28), the tumour sizes were 6.9 × 10^9^ ± 1.5 × 10^9^ (naïve), 3.5 × 10^9^ ± 1.6 × 10^9^ (L-ALD), 5.3 × 10^9^ ± 1.0 × 10^9^ (γδ T cells), and 2.1 × 10^9^ ± 8.2 × 10^8^ (L-ALD + γδ T cells) photons (Fig. 7). Although monotherapy of L-ALD or γδ T cells resulted in some tumour growth delay, only the combination treatment demonstrated a significant reduction in tumour growth (*p* = 0.015) with a ~ 3-fold decrease in tumour growth.

### 3.10 IFN-γ detected in sera of mice treated with γδ T cells and L-ALD

In order to determine whether L-ALD had activated of γδ T cells *in vivo*, the release of IFN-γ from the γδ T cells was measured. Analysis of sera samples from mice demonstrated detectable levels of human IFN-γ in the sera of the combinatory group only (9.2 ± 5.1 pg/ml). In the case of mice treated with γδ T cells alone, an insufficient amount of human IFN-γ was released to be detected.

## 4 Discussion

N-BPs have been shown to effectively sensitise various cancer types to Vγ9Vδ2 T cells in both preclinical [19], [21], [22], [23], [24], [25], [26] and clinical studies [44], [45], [46], [47]. Due to the known limitations of the pharmacokinetics of bisphosphonates [28], encapsulating these agents within liposomes offers an attractive solution to increase delivery of bisphosphonates to non-osseous tumour sites. ZOL is the most potent of the N-BPs [48], [49] and is the most widely used bisphosphonate in γδ T cell immunotherapy studies. However, in a study by Shmeeda et al. [29], it was shown that while encapsulating ZOL in liposomes increased the amount of ZOL in tumours *in vivo*, mice unexpectedly died 5–7 days after treatment with this formulation. This toxicity was also reported by co-authors of this work [23]. In this study, an alternative bisphosphonate, ALD, in the liposomal formulation was used and did not result in mice death at a therapeutically efficacious dose in an intraperitoneal ovarian tumour model. As we have also shown in a pseudo-metastatic lung melanoma tumour model (Fig. 7), significant inhibition of tumour growth was observed when L-ALD was used in combination with γδ T cells. A liposomal formulation of ZOL that was shown to increase survival time of prostate tumour-bearing mice with no toxicity observed, has also been reported [50]. However, this formulation was composed of Egg PC, DSPE-PEG_2000_ and cholesterol and had also been exposed to freeze-drying. When comparing the results of this study to their own, Shmeeda et al., suggested that the use of use of Egg PC and freeze drying led to a less stable formulation, and this could be the reason for the discrepancy in toxicity *in vivo* work [29]. A hybrid nanoparticle-liposome formulation has also been prepared consisting of a calcium phosphate core to which ZOL could bind mixed with DOTAP/cholesterol/DSPE-PEG_2000_ liposomes [37]. These hybrid particles achieved a significant tumour weight inhibition of 45%, and while no *in vivo* toxicity tests were performed, no sudden mouse death was observed [38].

L-ALD has also been used *in vivo*, as a monotherapy for a murine breast cancer model [31]. However, while some tumour growth inhibition was observed this did not reach significance, similarly to what we observe in this work. This suggests that ALD does not reach sufficient concentrations in tumours to be therapeutically efficacious as a monotherapy, even when encapsulated in a liposomal formulation. L-ALD has also been used in the treatment of inflammatory conditions [32], [33], [34], [35]. The ability of ALD liposomes to deplete monocytes and macrophages has been shown to inhibit restenosis and endometriosis in a rat model [35]. This anti-inflammatory activity of ALD liposomes has shown to be effective in the inhibition of restenosis in rabbits *in vivo* [30]. These liposomes were negatively charged due to the inclusion of distearoyl-phosphatidylglycerol (DSPG), and had a zeta potential of approximately − 29 mV. Additionally, a clinical trial involving the use of L-ALD in coronary artery restenosis prevention is due to commence this year [36]. ALD has also been co-encapsulated with doxorubicin (DOX) into liposomes [39]. Liposomes encapsulating both drugs were shown to be more effective than liposomes encapsulating DOX alone at inhibiting tumour growth in 4T1 breast cancer and M109R lung cancer models in BALB/c mice *in vivo*. While γδ T cells were not used in this study, the *in vivo* toxicity of L-ALD was also examined with the incorporation of ALD in liposomes shown to lead to a 40 fold increase in IL-1β secretion from monocytes *in vitro,* but did not activate the complement system in human plasma [39]. Although L-ALD has been assumed to be safe substitute for L-ZOL, comparative *in vivo* toxicity studies have not been performed. In the current report, we examine the ability of L-ALD to substitute L-ZOL, as a γδ T cells sensitiser *in vitro*, followed by conducting a comparative *in vivo* toxicity study for both formulations after single i.v. injection, at therapeutically relevant doses in mice. *In vivo* L-ALD toxicity, following multiple i.v. injections, mimicking the immunotherapy therapy protocol, was also assessed. Organ biodistribution studies of empty and N-BP loaded liposomes were performed in order to help partially understand findings of the *in vivo* comparative toxicity study.

ALD and ZOL are second and third generation N-BPs, respectively [51]. In our studies we have seen that ZOL is ~ 5 times more potent as both a monotherapy and as a sensitising agent for γδ T cell immunotherapy. Similar findings in relation to the potency of the two N-BPs have been reported in the literature. ZOL had IC_50_ values of 0.02 ± 0.00 μM for inhibition of FPP synthase in J774 cell homogenates and 0.003 ± 0.000 μM for inhibition of recombinant human FPP synthase. ALD, however had IC_50_ values of 0.50 ± 0.15 μM and 0.05 ± 0.001 μM, respectively. The necessity to use increased concentrations of L-ALD compared to L-ZOL is consistent with these findings. A study that compared four different formulations of L-ZOL on their cytotoxic ability found that unless the liposomes were targeted to the folate receptor, a reduction in cell viability was not observed at concentrations up to 200 μM [52]. However, this study was looking at the direct cytotoxic action of ZOL as a monotherapy. Much lower concentrations of ZOL are required to sensitise cancer cells to γδ T cells hence in our study we did not explore the active targeting approach and much lower concentrations of ZOL were used. L-ALD however seems to exhibit higher IC50 values than L-ZOL so utilisation of active targeting approach for this type of formulation in the future is worth investigating, to establish if lower L-ALD doses, which are more relevant for *in vivo* settings, can be used. Co-authors of this work have previously studied the ability of L-ZOL and L-ALD to sensitise the ovarian cancer cell line IGROV-1 to destruction by γδ T cells *in vitro* [23]. L-ZOL (0.1 μg/ml, ~ 0.25 μM) and L-ALD (0.2 μg/ml, ~ 0.6 μM) led to ~ 25% apoptotic cells and ~ 30% reduction in cell viability when used in combination with γδ T cells, at much lower concentrations than used in our study (3–10 μM and 40–60 μM for L-ZOL and L-ALD, respectively). These are much lower concentrations than used in our study (3–10 μM and 40–60 μM for L-ZOL and L-ALD, respectively) but cell lines used are also different.

In the present study, and for the first time, we directly compared the *in vivo* toxicity of L-ZOL and L-ALD. Based on the results of the *in vitro* assays, L-ALD were used at a concentration five times higher (0.5 μmol/mouse) than that of L-ZOL (0.1 μmol/mouse). The dose of L-ALD used matches that used in the study by Parente-Pereira et al. [23]. Shmeeda et al. assessed the toxicity of L-ZOL in BALB/c and Sabra mice [29], and it was shown that L-ZOL resulted in splenomegaly (~ 200 mg *vs.* 120 mg/spleen) and leucocytosis (~ 30 × 10^3^ WBC/μl *vs.* ~ 5 × 10^3^ WBC/μl). Despite the different stain of mice used in this study, similar increases in spleen weight (0.06 ± 0.02 g *vs.* 0.03 ± 0.004 g) and WBC concentration (3.22 ± 2.49 10e^9^/L *vs.* 0.77 ± 0.15 10e^9^/L) were observed (Fig. 7). Macrophage depletion, as a result of liposomal BPs administration, has been reported to lead to splenomegaly [53]. Additionally there were several differences between the result reported here and the results of Shmeeda et al., with no reduction in platelet number or haemoglobin observed in our study unlike their previous report [29]. Different time points at which the mice were sacrificed post-injection were used (3 days *vs.* 5 days, respectively). Consistent to what we report here, the toxicity of L-ALD has been shown to be non-cumulative after multiple injections by Gabizon and co-workers [39]. Despite the comparable *in vivo* toxicity profiles of L-ALD and L-ZOL, L-ALD and L-ZOL resulted in 100% and 0% mice survival, respectively, at the studied doses. NSG mice are not the best models to carryout toxicity studies for two reasons; they are known to have several defects in cytokine signalling pathways [54]. Secondly, it was previously hypothesised that the mechanism of *in vivo* L-ZOL toxicity is linked to cytokine release from macrophages [29]. It is therefore interesting to observe comparable L-ZOL toxicity profiles in both immunocompetent and immunocompromised mice. Such puzzling results cannot be explained using the results presented here. A previous study by Parente-Pereira et al. [23] used the same dosage regimen of L-ALD and showed no mice death. However, no toxicity profiling was carried out in that study. Different markers for *in vivo* toxicity may need to be assessed in order to be able to differentiate between the toxicity of L-ZOL and L-ALD. For example, in rat and rabbit models, complement and IL-2β markers after injection of L-ALD have been studied *in vivo* [55]. L-ALD led to increased secretion of IL-2β in both rat and rabbit, with minor complement activation seen in rat only. However, the liposomes in these studies were more negatively charged that those used in our study, which may lead to differences in *in vivo* toxicity. Similar studies have not been undertaken for L-ZOL.

The formulation of the liposomes can also influence their *in vivo* behaviour. In this study, the size of the liposomes is within the range (100–200 nm) reported to be extravasated in regions of leaky vasculature as part of the EPR effect [56] and the low PDI values indicate that the liposomes are homogenous. The L-ZOL and L-ALD obtained have similar physicochemical characteristics and drug loading, allowing for direct comparisons to be made between them (Table S1). In order to better understand the results of the *in vivo* toxicity studies, the biodistribution of EL, L-ZOL and L-ALD in tumour bearing mice was studied. A three-fold increase in the spleen uptake of both L-ALD and L-ZOL was observed compared to EL. The increase in spleen uptake of L-ALD in comparison to liposomal doxorubicin has been previously reported [39]. The spleen uptake of L-ALD has not been compared to L-ZOL previously and it was not known whether a difference in spleen uptake of the two formulations could account for the increased toxicity of L-ZOL. Our results confirmed no significant differences in spleen uptake between the uptake of L-ZOL and L-ALD. Such results at least concluded that the sudden mice death in case of L-ZOL was not due to this higher uptake in spleen. The increased spleen uptake may be as a result of the well-reported macrophage depletion properties of liposomal N-BPs [32], [57], whereby macrophages that uptake L-ZOL or L-ALD undergo macrophage apoptosis [58]. This would result in the trafficking of damaged macrophages containing L-ZOL or L-ALD to the spleen [59], which also explains the higher levels of radioactivity detected in the spleen than EL (Fig. 6). Furthermore, this may account for the significant increase in spleen weight observed in mice injected with L-ZOL or L-ALD (Fig. 5). Interestingly, despite the higher accumulation of L-ZOL or L-ALD in the spleen no histological changes were observed in the spleen tissues. The biodistribution of L-ZOL has previously been reported [29], but empty liposomes were not used as a control in this study. Once again, however, no discernible differences between L-ZOL and L-ALD were observed that may account for the differences in toxicity of these two formulations. Liposomal formulations of drugs were originally proposed to reduce systemic toxicity of drugs. The increased toxicity of ZOL when it was encapsulated into liposomes contrasts the original purpose of its nanoformulation and highlights the importance of considering the free drug and its nanoformulation as two separate entities.

In our study, combinatory γδ T cell immunotherapy was shown to significantly reduce tumour growth in a pre-clinical mouse model. Mice treated with L-ALD and γδ T cell showed a ~ 3-fold decrease in tumour growth when compared to naïve mice (*p* > 0.05). L-ALD has not yet been used in combination with γδ T cells in clinical studies. Only one study has been done on the use of L-ALD in combination with human γδ T cells, by co-authors of this work [23]. In the reported study, Parente et al. used L-ALD in combination with human-derived γδ T cells to treat an intraperitoneal ovarian cancer model in mice [23]. Significant reductions in tumour growth was observed in mice injected with both L-ALD and γδ T cells. Our study utilised the same L-ALD and γδ T cells doses reported by Parente et al., but the route of γδ T cells administration was different; Parente et al. injected γδ T cells intraperitoneally while in our study cells were injected intravenously. Both studies however concluded that L-ALD was necessary to improve the potency of γδ T cells immunotherapy.

Clinically, γδ T immunotherapy has been used for the treatment of renal cell carcinoma [7], [8], [45], multiple myeloma [6], non-small cell lung cancer [11], [60] and other various solid tumours [9], [10], with disease stabilisation achieved in the majority of these studies. However, N-BPs were not used in most of these studies, suggesting that the full potential of γδ T immunotherapy has yet to be explored. While there is a clinical trial for L-ALD due to commence as a monotherapy [36], its use as a sensitiser for γδ T immunotherapy in cancer has yet to be assessed in humans. It is also possible that L-ALD may also have anti-cancer activities unrelated to γδ T cell sensitisation. A direct cytotoxic effect may not have a significant therapeutic effect at the dose used. However, the ability of L-ALD to lead to monocyte and macrophage depletion has been well reported and has shown benefits in the treatment of restenosis and endometriosis [55], [61]. This aspect of L-ALD activity may help contribute to its anti-cancer properties as high levels of macrophages in the tumour have been associated with disease progression and treatment resistance [62].

## 5 Conclusion

While some toxic side effects were seen after injection of L-ALD, namely increased spleen weight, leucocytosis, neutrophilia and lymphocytopenia, mice injected with L-ALD had a 100% survival rate while L-ZOL resulted in mice death. Despite L-ALD being ~ 5 times less potent than L-ZOL at sensitising tumour cells to destruction by γδ T cells *in vitro*, it is evident from the *in vivo* therapy study that therapeutically relevant concentrations of L-ALD and γδ T cells could be achieved in tumour tissues, following systemic administration. L-ALD has been shown to be efficacious as a sensitiser for γδ T cell immunotherapy, and the combinatory therapy resulted in activation of γδ T cells and delayed tumour growth in an experimental metastatic lung mouse model.

## Figures and Tables

**Table 1:** Haematological results^a^ from male non-tumour bearing NSG mice treated with a single dose of 0.1 μmol L-ZOL or 0.5 μmol L-ALD and sampled 72 h after dosing^b^.

	Control^c^		L-ZOL^c^		L-ALD^c^	
	Mean ± SD	Range	Mean ± SD	Range	Mean ± SD	Range
WBC	0.8 ± 0.2	0.6–1.0	3.2 ± 2.5⁎⁎	1.3–3.2	2.2 ± 0.5⁎⁎⁎	1.7–2.8
Neutrophils	0.5 ± 0.1	0.4–0.7	3.0 ± 2.5⁎⁎	1.3–2.8	2.0 ± 0.5⁎⁎⁎	1.5–2.6
Neutrophils %	66.2 ± 7.9	66.0–76.0	92.2 ± 4.3⁎⁎⁎	87.0–97.0	88.6 ± 3.7⁎⁎⁎	83.0–91.0
Lymphocytes	0.2 ± 0.1	0.1–0.2	0.1 ± 0.1	0.0–0.2	0.1 ± 0.1	0.0–0.2
Lymphocytes %	21.0 ± 10.4	10.0–22.0	3.0 ± 1.7⁎⁎	2.0–6.0	5.2 ± 2.2⁎	2.0–8.0
Monocytes	0.1 ± 0.1	0.0–0.2	0.1 ± 0.1	0.0–0.1	0.1 ± 0.1	0.1–0.2
Monocytes %	10.6 ± 7.9	3.0–23.0	3.8 ± 3.0	1.0–8.0	6.0 ± 3.4	3.0–11.0
Eosinophils	0.0 ± 0.0	0.0–0.0	0.0 ± 0.0	0.0–0.0	0.0 ± 0.0	0.0–0.0
Eosinophils %	2.2 ± 1.3	0.0–3.0	1.0 ± 1.2	0.0–3.0	0.2 ± 0.4⁎	0.0–1.0
Basophils	0.0 ± 0.1	0.0–0.1	0.0 ± 0.0	0.0–0.0	0.0 ± 0.0	0.0–0.0
Basophils %	3.7 ± 5.1	0.0–9.8	0.0 ± 000	0 0.0–0.0	0.0 ± 0.0	0.0–0.0
RBC	7.7 ± 0.6	7.1–8.7	8.0 ± 0.4	7.4–8.4	7.9 ± 0.5	7.3–8.4
HGB	13.0 ± 1.2	12.1–14.8	13.5 ± 0.7	12.6–14.3	12.7 ± 0.6	12.0–13.3
HCT	43.0 ± 4.3	38.2–49.1	44.2 ± 1.6	42.2–45.6	42.0 ± 2.4	38.8–44.3
MCV	56.1 ± 1.9	53.4–58.7	55.3 ± 1.5	53.6–57.6	52.9 ± 0.5⁎⁎	52.6–53.7
MCH	17.0 ± 0.4	16.2–17.3	16.9 ± 0.4	16.4–17.5	16.0 ± 0.3⁎⁎	15.7–16.3
MCHC	30.2 ± 1.0	29.4–31.7	30.5 ± 0.8	29.4–31.4	30.3 ± 0.4	29.9–30.8
RDW	14.6 ± 0.6	13.7–15.2	14.7 ± 0.4	14.0–15.0	14.6 ± 0.2	14.3–14.7
PLT	1331.0 ± 104.2	1179–144	1223.4 ± 194.2	1023–1501	1472. ± 145.7	1232–1572
PCV	35.4 ± 5.2	34.0–44.0	36.0 ± 2.7	32.0–39.0	35.4 ± 2.1	34.0–39.0

Student's *t-*test *vs.* naive.

a Values are means ± SD (*n* = 5).

b Abbreviations and units: WBC, white blood cell, 10e9/L; Neutrophils, 10e9/L; Lymphocytes, 10e9/L; Monocytes, 10e9/L; Eosinophils, 10e9/L; Basophils, 10e9/L; RBC, red blood cells, 10e12/L; HGB, haemoglobin, g/dL; HCT, haematocrit, %; MCV, mean cell volume, fL; MCH, mean cell haemoglobin, pg; MCHC, mean cell haemoglobin concentration, g/dL; RDW, rec cell distribution width, %; PLT, platelets, 10e9/L; PCV, packed cell volume, %.

c Data was expressed as means ± SD (*n* = 5).

⁎ *p* < 0.05.

⁎⁎ *p* < 0.01.

⁎⁎⁎ *p* < 0.001.

## Appendix A Supplementary data

Supplementary material Image 2
