# Supplementary material for: In vitro potency, in vitro and in vivo efficacy of liposomal alendronate in combination with γδ T cell immunotherapy in mice
Source: J Control Release. 2016 Nov 10;241:229–41. doi: 10.1016/j.jconrel.2016.09.023 (PMC5073077; doi:10.1016/j.jconrel.2016.09.023)
Supplement: Supplementary file 1 — Supplementary material [file mmc1.pdf]

In vitro potency, *in vitro* and *in vivo* efficacy of liposomal alendronate in combination with γδ T cell immunotherapy in mice

*Naomi O. Hodgins, Wafa' T. Al-Jamal, Julie T-W. Wang, Ana C. Parente-Pereira, Mao Liu, John Maher and Khuloud T. Al-Jamal**

N.O. Hodgins, Dr. J. T-W. Wang, M. Liu, Dr. K.T. Al-Jamal
King's College London
150 Stamford Street
Institute of Pharmaceutical Science
Franklin-Wilkins Building
150 Stamford Street
London SE1 9NH, UK

Dr. W.T. Al-Jamal
School of Pharmacy,
University of East Anglia, Norwich Research Park
Norwich NR4 7TJ, UK

Dr. A.C. Parente-Pereira, Dr. J. Maher
King's College London
Division of Cancer Studies
Guy's Hospital
London SE1 9RT

* Corresponding author - E-mail: khuloud.al-jamal@kcl.ac.uk, john.maher@kcl.ac.uk

Supplementary Materials

Human AB serum (male), ferric chloride hexahydrate, ammonium thiocyanate and copper (II) sulphate pentahydrate were purchased from Sigma (UK). Roswell Park Memorial Institute medium (RPMI) was purchased from Invitrogen (UK). Acetonitrile (HPLC grade) and tetra-n-butyl ammonium hydrogen sulphate were obtained from Fisher (UK). Sodium hydroxide and perchloric acid were obtained from AnalaR NORMAPUR (UK). O-phthalaldehyde (OPA) was obtained from Apollo Scientific Limited (UK). 2-mercaptoethanol (2ME) was obtained from VWR international (UK). Di-sodium hydrogen orthophosphate was obtained from BDH chemicals (UK). Ficoll-Paque Plus was purchased from GE Healthcare (UK). Citrate-dextrose Solution was purchased from SLS (UK). IL-2 (100U/ml) (Proleukin®) was obtained from Prometheus (USA). T Cell Receptor (TCR) Pan gamma/delta-FITC and IgG FITC Isotype controls were purchased from Beckman Coulter (UK).

Supplementary Methods

Preparation of liposomes

Lipid stock solutions were prepared in chloroform/methanol (4:1 v/v) at concentrations of 20-40 mg/ml. Lipid solutions were stored at -20°C under nitrogen to avoid degradation. Empty liposomes (EL), zoledronic acid liposomes (L-ZOL) and alendronic acid liposomes (L-ALD) were prepared [1] using one of these two methods:

Thin-Film Hydration (TFH)

The details of the method are described in the main paper under methods section.

Reverse Phase Evaporation (RVE)

DSPC, cholesterol and DSPE-PEG2000 (55:40:5 molar volume) were transferred to a 25 ml round-bottom flask and 2 ml chloroform/methanol (4:1 v/v) was added. A lipid film was

formed by removing the solvent under reduced pressure by a rotary evaporator. The lipid film was then re-dissolved in 3 ml diethyl ether and 3 ml chloroform. The aqueous phase (HBS, 1.5 ml) was then added and the solution was emulsified by sonicating for 10 min at 60°C in a bath-type sonicator (Ultrasonic Cleaner, VWR). The resulting emulsion was placed on the rotary evaporator to remove the organic solvent under reduced pressure. Evaporation continues until a gel is formed; further evaporation causes spontaneous formation of liposomes and subsequently ensures that all traces of the organic solvent have been removed. A final volume of 1 ml liposome suspension was recovered and stored at 4°C.

Physicochemical characterisation of liposomes

The hydrodynamic diameter, polydispersity index and zeta potential of the liposomes were measured using the NanoZS (Malvern Instrument, UK). Hydrodynamic size, polydispersity index and Zeta potential were measured in disposable square polystyrene cuvettes and disposable capillary cells, respectively (Malvern Instrument, UK). The original sample (20 µl) was diluted to 1.5 ml with 10 mM sodium chloride. The measurements were carried out at 25°C. Three measurements were performed and the mean and standard deviation were calculated for each sample.

Lipid recovery quantification

Stewart's assay was used to determine the lipid concentrations before and after purification with size exclusion chromatography [1]. Stewart's reagent (0.1 M ammonium ferrothiocyanate solution) was prepared using 5.46 g ferric chloride hexahydrate and 6.08 g of ammonium thiocyanate and made up to 200 ml with deionised water. The solution to be measured was prepared by mixing 50 µl of the unknown sample (or the standard), 2 ml of chloroform and 2 ml of Stewart's Reagent. The mixture was centrifuged at 1000 g in a bench

centrifuge (Centrifuge 5810 R, Eppendorf) for 10 min and the organic layer was removed and analysed with UV spectrometer (Lambda 35, Perkin Elmer). The absorbance at 485 nm was used to determine the lipid concentration. Calibration curves were prepared in the same way using known amounts of lipid as standards. The lipid recovery was determined by comparing the lipid concentration of a liposome sample before and after purification.

Quantification of ZOL

Two different methods were used to quantify ZOL in liposomal formulations; Reverse Phase High Performance Liquid Chromatography (RP-HPLC) [2] with UV spectroscopy, or UV detection alone. UV spectroscopy alone was used to determine the percentage encapsulation efficiency (% EE) of the liposomes. RP-HPLC with UV spectroscopy was used in release studies since a method with greater sensitivity was required.

Sample processing and ZOL standard curves

A calibration curve containing 5 mM empty liposomes and known concentrations of free ZOL, referred to 'ZOL spiked liposomes' samples, were prepared. ZOL concentrations ranged between 0.1-1 mM and 40-400 µg/ml (2-20 µg per 50 µl injection volume) for samples quantified with UV spectroscopy or RP-HPLC, respectively. A calibration curve containing free ZOL at the same concentration range was prepared to ensure that the presence of lipid did not interfere with the measurements. L-ZOL samples to be quantified (or standards) were both processed using the Folch method prior to quantification. This method disrupts the liposomes; allowing the encapsulated ZOL to be released into the aqueous phase and separating any hydrophobic components (cholesterol, lipids etc.) from the hydrophilic drug. In brief, chloroform and methanol were added to a sample of liposomes at 8:4:3 (chloroform: methanol: liposome suspension) volume ratio. The sample was then vortexed (Vortex genie 2, Scientific Industries Inc, USA) and centrifuged at 10000 rpm for 10 minutes

(Centrifuge 5810 R, Eppendorf). Two layers were formed after centrifugation and the upper aqueous layer containing the ZOL was removed and quantified using one of the two methods described below:

UV spectroscopy

A 0.5 ml sample of the upper aqueous phase was added to 0.5 ml of DI water. The samples were then read for absorbance at 210 nm with UV spectrometer (Lambda 35, Perkin Elmer), against a HBS reagent blank. Concentrations were calculated from the 'ZOL spiked liposomes' calibration curve.

RP-HPLC with UV spectroscopy

A 0.5 ml sample of the upper aqueous phase was transferred to an HPLC vial for analysis.

The Jasco HPLC instrument was used with a Jasco PU-2089 Plus pump, Jasco CO-2067 Plus oven, Hasco UV-2075 Plus UV/Vis Detector and Jasco AS-2050 Plus Sampler. The quantitative analysis of ZOL was performed on a Gemini C18 column (150x4.60 mm; 5 µ; 110 Å; Phenomenex UK). The mobile phase consisted of an aqueous buffer (8 mM di-potassium hydrogen orthophosphate, 2 mM di-sodium hydrogen orthophosphate and 7 mM tetra-n-butyl ammonium hydrogen sulphate adjusted to pH 7.0 with sodium hydroxide) and acetonitrile (85:15). The mobile phase was filtered through a 0.2 µm membrane filter and degassed by sonication (0.5 hr/L) before use. The flow rate was 1.0 ml/min with isocratic conditions used. The temperature of the column was 35°C. The wavelength of the UV/Vis detector was set at 210 nm. Concentrations were calculated from the 'ZOL spiked liposomes' calibration curve.

Quantification of ALD

ALD concentrations, for determination of % EE or percentage drug released, were determined with a copper sulphate-based UV spectroscopy method [3] or o-phthalaldehyde (OPA)-based fluorescence method [4], respectively.

Sample processing and ALD standard curves

A calibration curve containing 5 mM empty liposomes and known concentrations of free ALD, referred to 'ALD spiked liposomes' samples, was prepared. ALD concentrations ranged between 0.1-1 mM and 0.5-5 µM for samples quantified with copper sulphate-based UV detection method or o-phthalaldehyde (OPA)-based fluorescence method, respectively.

L-ALD samples to be quantified (or standards) were both processed using the Folch method prior quantification as described above for ZOL. The upper aqueous layer containing the ALD was removed and quantified using one of the two methods described below:

Quantification using copper sulphate

$CuSO_4$ reagent was prepared by dissolving 10 mM $CuSO_4$ in deionised water. A 0.5 ml sample of the upper aqueous phase was added to 0.5 ml of 10 mM $CuSO_4$ reagent. After 10 minutes, the UV absorbance at 240 nm was measured with a UV/Vis Spectrophotometer (Perkin Elmer, Model: Lambda 35), using HBS as the reagent blank. Concentrations were calculated from the 'ALD spiked liposomes' calibration curve.

Quantification using OPA/ 2ME reagent

OPA/2ME reagent was prepared using 10 mg of OPA, 50 µL of 2ME, with the volume completed to 10 mL using 0.05 M NaOH. A 0.2 ml sample of the upper aqueous layer to be assessed for ALD was mixed with 0.1 mL of OPA/2ME reagent and the volume was completed to 2 ml with 0.05 M NaOH. The emission intensity was recorded between 380-600 nm at 360 nm excitation wavelength using a luminescence spectrophotometer (Perkin Elmer,

Model: LS50B). The absorbance was read at 450 nm emission for all samples. Concentrations were calculated from the 'ALD spiked liposomes' calibration curve.

Quantification of N-BP encapsulation efficiency (%EE) and drug loading

The drug loading and encapsulation efficiency was quantified with UV spectroscopy (ZOL), copper sulphate and UV spectroscopy (ALD) and Stewart's assay (lipid). The amount of drug entrapped within liposomes was quantified for this purpose. A sample of the liposomes was taken and processed using the Folch method, the N-BP in the upper aqueous layer was then quantified with RP-HPLC (210 nm) (ZOL) or OPA method (ALD), as described above. Encapsulation Efficiency (EE %) was expressed as the percentage of N-BP loaded from the initial amount used, taking into account dilution factors. Drug loading was expressed as N-BP's μmol per lipid's μmol in the purified liposome sample. The quantity of N-BP in each liposome sample was measured three times and expressed as mean ± standard.

Liposome release studies

Drug release was carried out using the dialysis method [1]. One millilitre containing L-ZOL, L-ALD, or the free drug as controls (~5 μmol), was placed inside a 10 kD MWCO dialysis bag, in the presence or absence of 50% FBS, and dialysed against 200 ml HBS at 37°C under sink condition. Samples were obtained from inside (50 μl, L-ZOL) or outside (3 ml, L-ALD) the dialysis bag at different time points (t = 0.25, 0.5, 1, 2, 4, 8 and 24 h) and replaced with fresh HBS. ZOL and ALD contents were assessed with RP-HPLC (210 nm) and OPA method, respectively, as described above. L-ZOL samples had to be taken from inside the dialysis bag due to sensitivity limits of the detection method, whereas in the case of L-ALD the detection method used was sufficiently sensitive to allow samples to be taken from outside the dialysis bag. Percentage release was quantified by measuring the change in ZOL or ALD concentration inside or outside the dialysis bag, respectively.

Results were expressed as mean ± standard. Each experiment was performed in triplicate.

Isolation and expansion of γδ T cells

Blood samples of 20-30 ml were obtained from healthy volunteers. Ethical approval, "Use of Donor Blood Samples for Pre-Clinical Development of Active and Passive Immunotherapy for Cancer" (Ref.09/H0804/92) was obtained. The blood sample was added to 5 ml of Citrate-dextrose solution to prevent clotting. The sample was then layered on top of 15 ml of Ficoll-Paque Plus and centrifuged at 1150 g for 25 min, with no acceleration or breaks, using a bench centrifuge (Centrifuge 5810 R, Eppendorf). The layer of cells between the Ficoll-Paque Plus and the plasma was then removed and the resulting PBMCs were washed twice with PBS and were then suspended in RPMI 1640 (containing 10% human AB serum, 1% Glutamax and 1% antibiotic-antimycotic solution) at a concentration of $3x10^6$ cells/ml. In order to expand the γδ T cells, the PBMCs were activated with 1 µg/ml ZOL and 100 U/ml IL-2. Additional medium and 100 U/ml IL-2 were added every 2-3 days for 15 days.

Flow cytometry analysis of γδ T cells

On Day 1 and Day 15, 200 µl samples of the cell suspension were taken and 5 µl of either T Cell Receptor (TCR) Pan γ/δ-FITC antibody or IgG1 FITC Isotype control antibody was added. The cells were incubated with the antibodies for 20 min at 4°C before 1 ml PBS was added. Cells were centrifuged at 1000 rpm for 5 min in a bench centrifuge (Centrifuge 5810 R, Eppendorf). The supernatant was discarded and the cell pellet was re-suspended in 500 µl of PBS. All flow cytometric data were acquired using a Beckman Coulter Cytometer FC 500 MPL and were analysed using CXP Analysis software (Beckmann Coulter). The lymphocyte cell population was gated and the number of cells in this gate that express the γδ TCR were calculated as a percentage of the total lymphocytes.

Cell Viability Equation

$$\% \text{ Cell survival} = \frac{(\text{A570 nm of treated cells} - \text{A630 nm of treated cells})}{(\text{A570 nm of untreated cells} - \text{A630 nm of untreated cells})} \times 100$$

SUPPLEMENTARY RESULTS

L-ZOL and L-ALD of comparable size and drug loading were prepared

L-ZOL and L-ALD composed of DSPC:cholesterol:DSPE-PEG$_{2000}$ (55:40:2 molar ratio) were formulated using the Thin Film Hydration (TFH) and Reverse Phase Evaporation (RVE) methods (**Figure S1**). Liposomes were then extruded and purified using dialysis. All prepared formulations exhibited a hydrodynamic size of 155.4 – 159.0 nm, with narrow polydispersity index (PDI of 0.045-0.104) and slightly negative zeta potential (-11.7 to -14.0 mV). Overall, L-ZOL and L-ALD and empty liposomes (EL) with very similar characteristics were prepared using the TFH and RVE methods (**Table S1**).

To quantify the amount of ZOL and ALD encapsulated into liposomes, different quantification methods were developed, as described in the supplementary information. ZOL content was measured using UV-Vis and HPLC (**Figure S2**), while ALD encapsulation efficiency was determined using copper sulphate-based UV spectroscopy method and o-phthalaldehyde (OPA)-based fluorescence method (**Figure S3**). Our results showed that both ZOL and ALD had similar encapsulation efficiencies (% EE) ranging from 5.2 – 6.4%, with no significant differences between the TFH and RVE methods (**Table S2**). Drug loading of 0.23 – 0.27 mmol ZOL or ALD per mmol lipid was obtained ($p > 0.05$). As both preparation methods resulted in similar outcomes, TFH was adopted to formulate liposomes for all subsequent experiments, since it is less-time consuming.

3.2 L-ZOL and L-ALD showed low drug release in the presence of serum

In order to predict the *in vivo* stability of the liposomal formulations, we evaluated the drug release of L-ZOL and L-ALD at 37°C in both HBS and 50%. L-ZOL or L-ALD (or free drugs as controls) were suspended in HBS or 50% FBS and these preparations were placed in a dialysis bag with a 10 kD molecular weight cut off (MWCO). Dialysis was performed against HBS while maintaining sink conditions. Free ZOL or ALD was shown to readily exit the dialysis bag, with over 97% release by 4 h in all conditions, indicating that drug release was not impeded by the dialysis bag or the presence of serum (**Figure S4**).

In contrast to the free drugs, L-ZOL and L-ALD showed slower release profiles under similar conditions. In the absence of serum, L-ZOL and L-ALD showed release of ~ 2, 3 and 15% and 1, 5 and 5%, at 1, 8 and 24 h, respectively. In presence of serum, these values were ~ 2, 12 and 27% ($p < 0.05$) and 3, 11 ($p< 0.05$) and 17% ($p< 0.05$), respectively (FBS *vs.* HBS). It was concluded that there was a significant, but nonetheless slight (<10%) increase in drug release from the liposomes, when incubated with FBS. In light of the satisfactory stability of these formulations, we proceeded to *in vitro* or *in vivo* testing of their anti-tumour activity.

% EE and drug loading of ZOL in liposomes

A calibration curve of ZOL, in the range 0.1-1 mM, was achieved using the UV method, with no interference from liposomal components observed. This method allowed for quick and simple measurement of ZOL and was sensitive enough for determination of ZOL % EE in L-ZOL. However, for drug release studies, a more sensitive method was required. RP-HPLC was able to measure ZOL at concentrations as low as 40 µg/ml. Chromatograms of empty liposomes, ZOL, ZOL spiked liposomes and L-ZOL are shown in **Figure S2A**. ZOL showed retention times of 6 minutes, and no interference from the lipid was experienced.

Additionally, ZOL spiked liposomes or ZOL encapsulated liposomes (ZOL) produced a peak at 6 minutes, matching the elution profile of free ZOL. A calibration curve using ZOL spiked liposomes was prepared, and was used for quantification of ZOL concentration in L-ZOL (**Figure S2B**).

% EE and drug loading of ALD in liposomes

Calibration curves for ALD were obtained in the range 0.1-1 mM and 0.5-5 µM using copper sulphate-based UV detection method or OPA-based fluorescence method, respectively (**Figure S3**). Linear relationships between ALD concentrations and UV absorbance or fluorescence intensities were observed. No interference from residual lipids or other reagents used in the sample preparation process was seen. The copper sulphate-based UV detection method was sufficiently sensitive to quantify ALD % EE in L-ALD but its limit of detection was above that required for drug release studies. The OPA-based fluorescence method was ~200 times more sensitive and was used to quantify ALD in drug release studies.

γδ T cells isolated and purity quantified following expansion

γδ T cells were obtained from whole blood from healthy volunteers (n=22). γδ T cells were selectively expanded from the peripheral blood mononuclear cells (PBMC) by addition of IL-2 and ZOL **(Figure S8)**. The percentage and number of γδ T cells increased dramatically over a 15 day period. On day 1, the donors had a γδ T cell population of 4.9 ± 5.9 % of their total lymphocyte fraction (0.8 – 28.3 %) and by day 15 this had increased to 86.0 ± 9.3 % (**Figure S9**). The number of γδ T cells also increased with approximately 96 fold expansion in cell number between day 1 and day 15.

REFERENCES

[1] R. New, Liposomes: A Practical approach, (1990).
[2] G. Salzano, M. Marra, M. Porru, S. Zappavigna, A. Abbruzzese, M.I. La Rotonda, C. Leonetti, M. Caraglia, G. De Rosa, Self-assembly nanoparticles for the delivery of bisphosphonates into tumors, International journal of pharmaceutics, 403 (2011) 292-297.
[3] M. Koba, K. Koba, L. Przyborowski, Application of UV-derivative spectrophotometry for determination of some bisphosphonates drugs in pharmaceutical formulations, Acta poloniae pharmaceutica, 65 (2008) 289-294.
[4] J. Ezzati Nazhad Dolatabadi, H. Hamishehkar, M. de la Guardia, H. Valizadeh, A fast and simple spectrofluorometric method for the determination of alendronate sodium in pharmaceuticals, BioImpacts : BI, 4 (2014) 39-42.

SUPPLEMENTARY TABLES AND FIGURES

Table S1: Physicochemical characteristics of liposomes.

	Method of Preparation	Size (nm) [a], [c]	PDI	Zeta Potential (mV) [a], [c]
EL	TFH	156.0 ± 0.1	0.045 ± 0.004	-11.7± 1.3
	RVE	155.4 ± 2.4	0.104 ± 0.021	-14.0 ± 1.9
L-ZOL	TFH	156.6 ± 0.9	0.051 ± 0.02	-12.5 ± 0.6
	RVE	156.3 ± 2.0	0.082 ± 0.021	-13.3 ± 0.9
L-ALD	TFH	159.03 ± 1.1	0.100 ± 0.017	-12.00 ± 1.5
	RVE	156.6 ± 0.5	0.024 ± 0.009	-11.7 ± 1.4

[a] Hydrodynamic diameter measured with dynamic light scattering.
[b] Analysed with electrophoretic light scattering using 10 mM NaCl.
[c] Data are represented as mean ± SD (n=3).

Table S2: Lipid recovery and N-BP content of liposomes

	Method of Preparation	Lipid Recovery (µmol) [a], [d]	Lipid Recovery (%) [a], [d]	N-BP Recovery (µmol) [b], [c], [d]	%EE [b], [c], [d]	Drug Loading (µmol BP/ µmol Lipid)
L-ZOL	TFH	22.4 ± 1.9	89.6 ±7.7	6.1 ± 2.0	6.8 ± 2.2	0.27 ± 0.08
	RVE	23.9 ± 0.1	95.7 ± 0.4	5.3 ± 2.1	5.5 ± 2.2	0.23 ± 0.09
L-ALD	TFH	20.9 ± 0.7	83.8 ± 2.9	5.2 ± 0.2	6.2 ± 0.2	0.26 ± 0.01
	RVE	24.1 ± 0.3	96.3 ± 1.2	7.4 ± 3.0	7.7 ± 3.1	0.27 ± 0.13

[a] Measured with Stewart's assay at 25 µmol starting lipid amount.
[b] Measured with RP-HPLC or copper complexation for ZOL and ALD, respectively.
[c] At 100 µmol N-BP amount (100 mM, 1 ml) prior to purification
[d] Data are represented as mean ± SD (n=3).

Table S3: Serum biochemistry results[a] from male non-tumour bearing NSG mice treated with a single dose of 0.1 µmol L-ZOL or 0.5 µmol L-ALD and sampled 72 h after dosing[b]

	**Control[c]**		**L-ZOL[c]**		**L-ALD[c]**	
	Mean ± SD	Range	Mean ± SD	Range	Mean ± SD	Range
Albumin	31.6 ± 1.7	30.0 – 34.0	28.5 ± 2.5	28.5 – 31.0	**27.6 ± 1.7****	25.0 – 29.0
Sodium	154.8 ± 1.3	153.0 – 156.0	156.3 ± 10.7	142.0 – 165.0	173.6 ± 22.8	154.0 – 210.0
Potassium	7.48 ± 0.8	6.2 – 8.1	8.6 ± 0.8	7.7 – 9.6	7.1 ± 0.6	6.2 – 7.8
Chloride	108.8 ± 1.1	107.0 – 110.0	111.5 ± 7.6	101.0 – 117.0	87.7 ± 43.84	95.0 –111.0
Calcium	2.6 ± 0.2	2.5 – 3.0	2.5 ± 0.2	2.3 – 2.8	2.4 ± 0.1	2.3 – 2.5
Inorganic phosphorous	4.1 ± 0.5	3.3 – 4.8	3.8 ± 0.5	3.5 – 4.5	3.6 ± 0.5	3.0 – 4.2
Urea	8.4 ± 1.0	6.9 – 9.1	**6.8 ± 0.7***	6.3 – 7.8	7.7 ± 1.0	6.9 – 9.4
Creatinine	47.2 ± 6.3	40.0 – 48.0	45.0 ± 1.6	43.0 – 77.0	42.6 ± 16.9	20.0 ± 61.0
Cholesterol	3.2 ± 0.4	2.9 – 3.6	2.9 ± 0.3	2.5 – 3.2	8.5 ± 12.6	2.3 – 3.4
Total Bilirubin	3.8 ± 2.2	0.0 – 5.0	3.3 ± 0.5	3.0 – 4.0	1.8 ± 1.1	0.0 – 3.0
ALT	42.8 ± 23.8	25.0 – 83.0	32.8 ± 6.1	26.0 – 40.0	34.8 ± 10.3	21.0 – 50.0
AST	121.8 ± 33.2	75.0 – 166.0	144.5 ± 19.2	128.0 – 154.0	149.3 ± 31.8	120.0 – 185.0
ALP	81.2 ± 16.3	57.0 – 102.0	75.5 ± 18.6	62.0 – 97.0	79.7 ± 33.6	41.0 – 101.0

a Values are means ± SD (n=5)

b Abbreviations and units: Albumin, g/L; Sodium, mmol/L; Potassium, mmol/L; Chloride, mmol/L; Calcium, mmol/L; Inorganic phosphorous, mmol/L; Urea, mmol/L; Creatinine, µmol/L; Cholesterol, mmol/L; Total Bilirubin, µmol/L; ALT, alanine transaminase, U/l; AST, aspartate transaminase, U/l; ALP, alkaline phosphatase, U/l.

c Data are expressed as mean ± SD (n=5) *p< 0.05, **p< 0.01, ***p< 0.001. (Student's t test).

Table S4: Haematological results[a] from male non-tumour bearing NSG mice treated with three doses of 0.5 µmol L-ALD at one week intervals and sampled 72 h after the final dosing[b]

	**L-ALD[c]**	
	Mean ± SD	Range
WBC	**1.6 ± 0.2*** **	1.4 – 1.8
Neutrophils	**1.3 ± 0.2*****	1.0 – 1.4
Neutrophils %	**79.8 ± 9.4***	67.0 – 90.0
Lymphocytes	0.1 ± 0.1	0.0 – 0.2
Lymphocytes %	**8.0 ± 5.9***	3.0 – 17.0
Monocytes	0.2 ± 0.1	0.1 – 0.3
Monocytes %	12.0 ± 5.6	5.0 – 19.0
Eosinophils	0.0 ± 0.0	0.0 – 0.0
Eosinophils %	0.6 ± 0.9	0.0 – 2.0
Basophils	0.0 ± 0.0	0. 0 – 0.0
Basophils %	0.0 ± 0.0	0.0 – 0.0
RBC	7.6 ± 0.4	7.1 – 8.1
HGB	11.7 ± 0.7	10.5 – 12.2
HCT	40.0 ± 1.8	37.2 – 41.3
MCV	**52.4 ± 0.8****	51.3– 53.4
MCH	**15.2 ± 0.3*****	14.8– 15.6
MCHC	29.1 ± 0.6	28.2 – 29.7
RDW	14.9 ± 0.5	14.4 – 15.6
PLT	1421.2 ± 226.6	1261.0 – 1848.0
PCV	31.2 ± 2.4	27.0 – 33.0

a Values are means ± SD (n=5)
b Abbreviations and units: Albumin, g/L; Sodium, mmol/L; Potassium, mmol/L; Chloride, mmol/L; Calcium, mmol/L; Inorganic phosphorous, mmol/L; Urea, mmol/L; Creatinine, µmol/L; Cholesterol, mmol/L; Total Bilirubin, µmol/L; ALT, alanine transaminase, U/l; AST, aspartate transaminase, U/l; ALP, alkaline phosphatase, U/l.
c Data are expressed as mean ± SD (*n*=5) $*p< 0.05$, $**p< 0.01$, $***p< 0.001$. (Student's *t* test).

Table S5: Serum biochemistry results[a] from male non-tumour bearing NSG mice treated with three doses of 0.5 µmol L-ALD at one week intervals and sampled 72 h after the final dosing[b]

	**L-ALD[c]**	
	Mean ± SD	Range
Albumin	30.4 ± 0.6	30.0 – 31.0
Sodium	155.4 ± 0.9	154.0 – 156.0
Potassium	**5.7 ± 0.3****	5.3 – 6.2
Chloride	**110.8 ± 0.8***	110.0 – 112.0
Calcium	2.6 ± 0.0	2.6 – 2.6
Inorganic phosphorous	**3.2 ± 0.3****	2.8 – 3.4
Urea	8.2 ± 2.0	6.8 – 11.7
Creatinine	**39.2 ± 4.4***	33.0 – 45.0
Cholesterol	**2.6 ± 0.1****	2.5 – 2.8
Total Bilirubin	2.4 ± 0.6	2.0 – 3.0
ALT	64.8 ± 9.9	55.0 – 79.0
AST	120.6 ± 12.0	105.0 – 138.0
ALP	**61.8 ± 6.2***	52.0 – 67.0

a Values are means ± SD (n=5)

b Abbreviations and units: Albumin, g/L; Sodium, mmol/L; Potassium, mmol/L; Chloride, mmol/L; Calcium, mmol/L; Inorganic phosphorous, mmol/L; Urea, mmol/L; Creatinine, µmol/L; Cholesterol, mmol/L; Total Bilirubin, µmol/L; ALT, alanine transaminase, U/l; AST, aspartate transaminase, U/l; ALP, alkaline phosphatase, U/l.

c Data are expressed as mean ± SD (n=5) *p< 0.05, **p< 0.01, ***p< 0.001. (Student's t test).

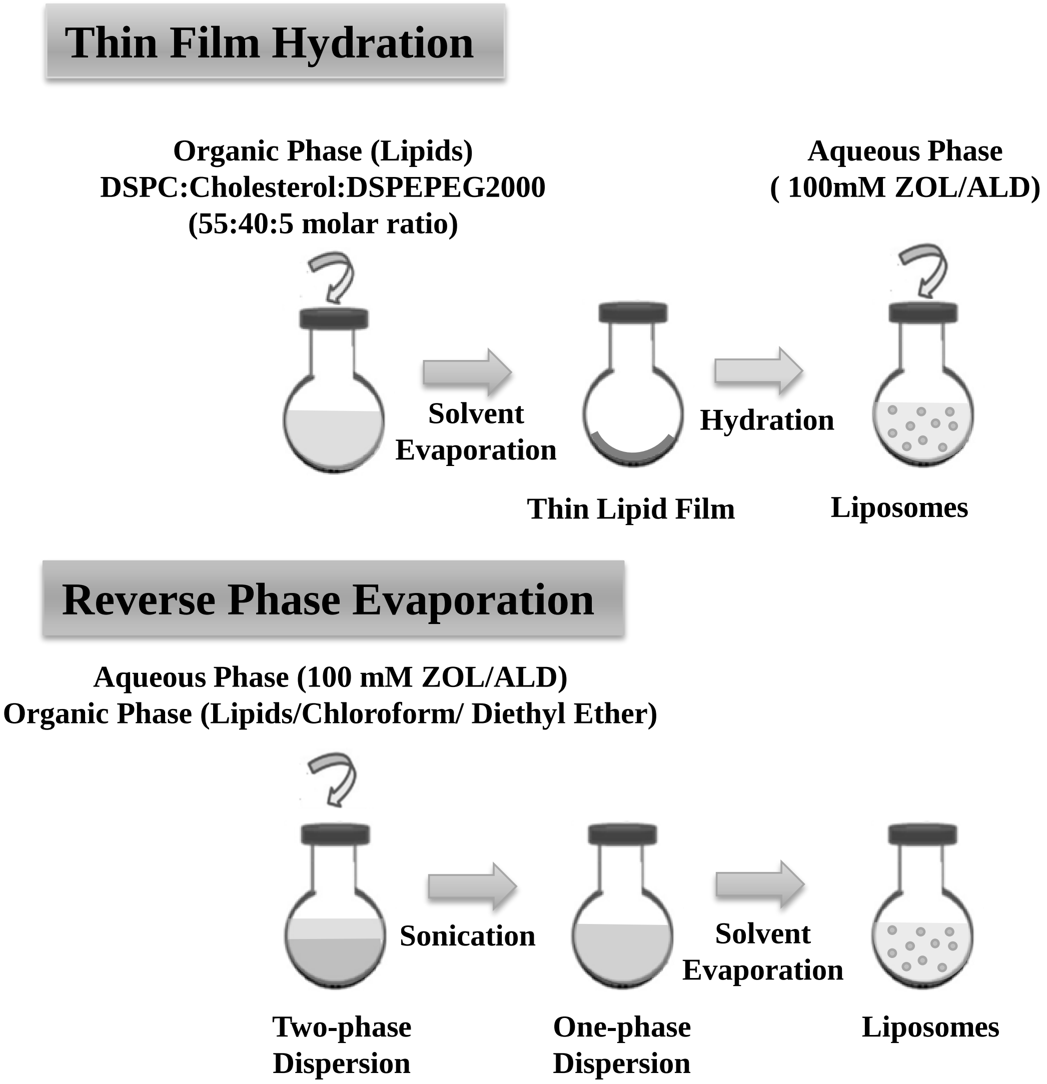

Figure S1: Two methods used for liposome formulation. Two different methods were used to formulate liposomes; thin film hydration (TFH) and reverse phase evaporation (RVE). For the TFH method, a lipid film was formed and was then hydrated with the aqueous phase. For the RVE method, the lipid film was used to form an emulsion with the aqueous phase, chloroform and diethyl ether. The organic solvents were then evaporated leading to the formation of the liposomes. The two methods produced liposomes with similar physicochemical properties and encapsulation efficiencies.

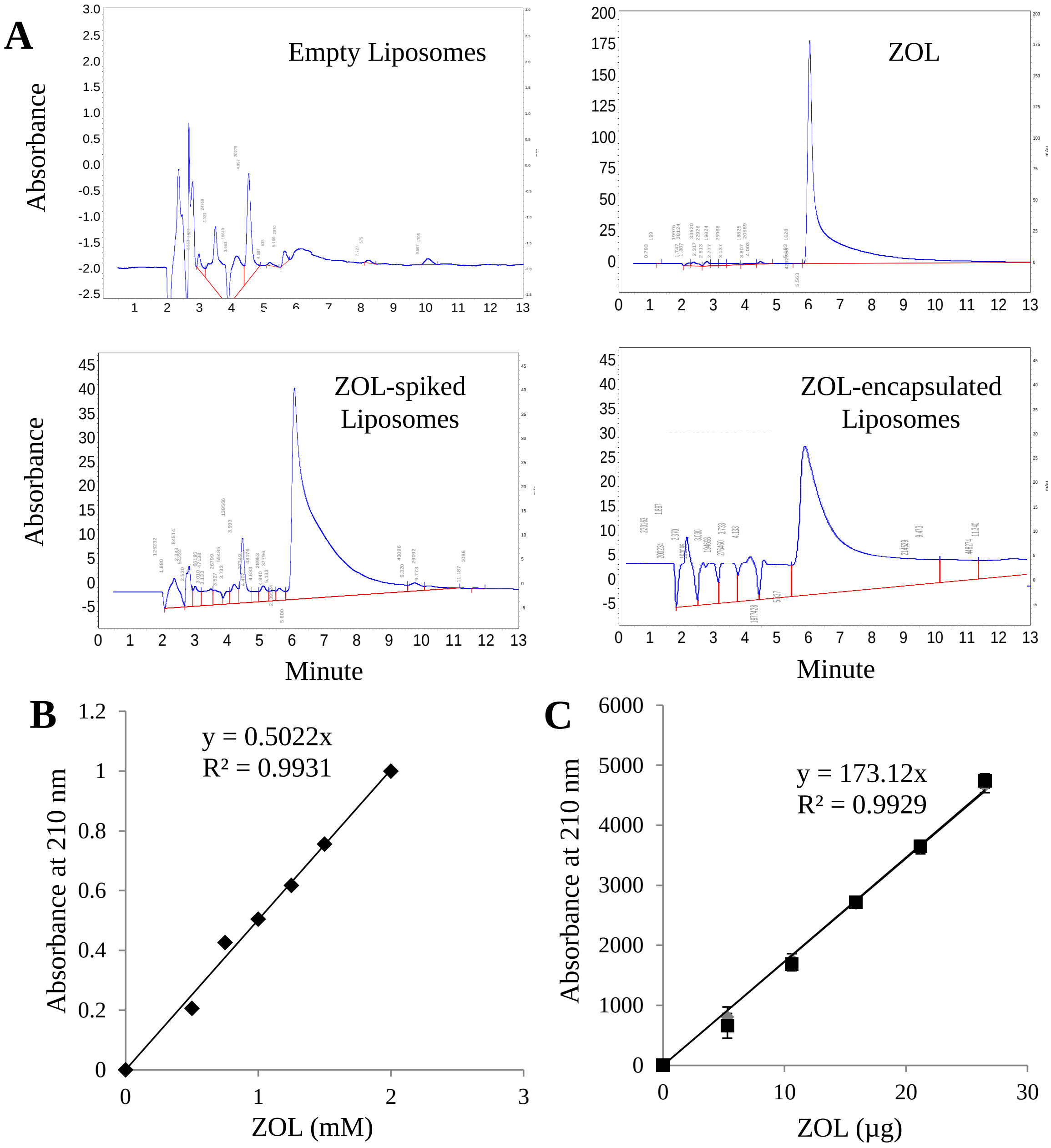

Figure S2: Quantification of ZOL using RP-HPLC with UV spectroscopy at 210 nm. (A) HPLC chromatographs showing the chromatograms of EL, free ZOL, or ZOL spiked or encapsulated into liposomes. The samples were prepared by adding chloroform to a portion of the liposomes to extract the lipid and release the ZOL into the aqueous phase. After centrifugation the aqueous layer was taken and analysed with RP-HPLC using a C18 column and a mobile phase of an aqueous buffer (8 mM di-potassium hydrogen orthophosphate, 2 mM di-sodium hydrogen orthophosphate and 7 mM tetra-n-butyl ammonium hydrogen sulphate adjusted to pH 7.0 with sodium hydroxide) and acetonitrile (85:15 v/v). UV detection at 210 nm was used. **(B)** Calibration curves of ZOL spiked liposomes using UV. **(C)** Calibration curves of ZOL and ZOL spiked liposomes using RP-HPLC indicating that the presence of liposomes does not interfere with the quantification of ZOL.

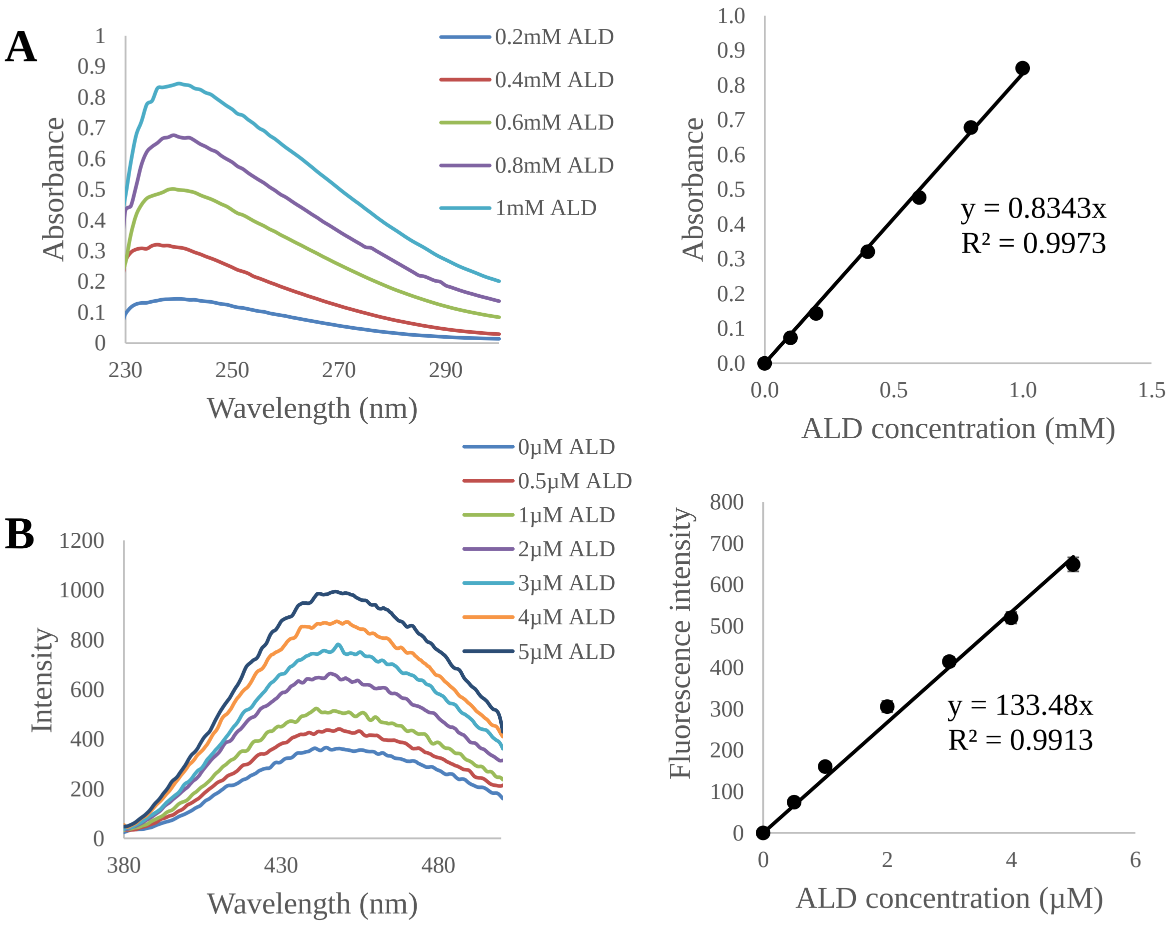

Figure S3: Quantification of ALD using two spectroscopic methods. Two different methods were used to quantify ALD. **(A)** The UV spectra and calibration curves obtained in the range 0.1 – 1 mM using Cu complexation method. UV spectroscopy was used to quantify the ALD content of liposomes, whereby ALD was complexed with copper. ALD was released from the liposomes using the Folch method and the aqueous layer was removed and added to an equal volume of 10 mM copper sulphate. The UV absorbance at 240 nm was then measured. **(B)** The spectra and calibration curves obtained in the range 1 – 5 µM using OPA fluorescence method. ALD was detected using fluorescence by conjugating ALD to o-phthalaldehyde (OPA) in order to quantify ALD released from the liposomes. The OPA reagent was prepared by using 10 mg of OPA, 50 µL of 2 - mercaptoethanol, with the volume completed to 10 mL using 0.05 M NaOH. The sample was excited at 360 nm and the emission was measured at 450 nm.

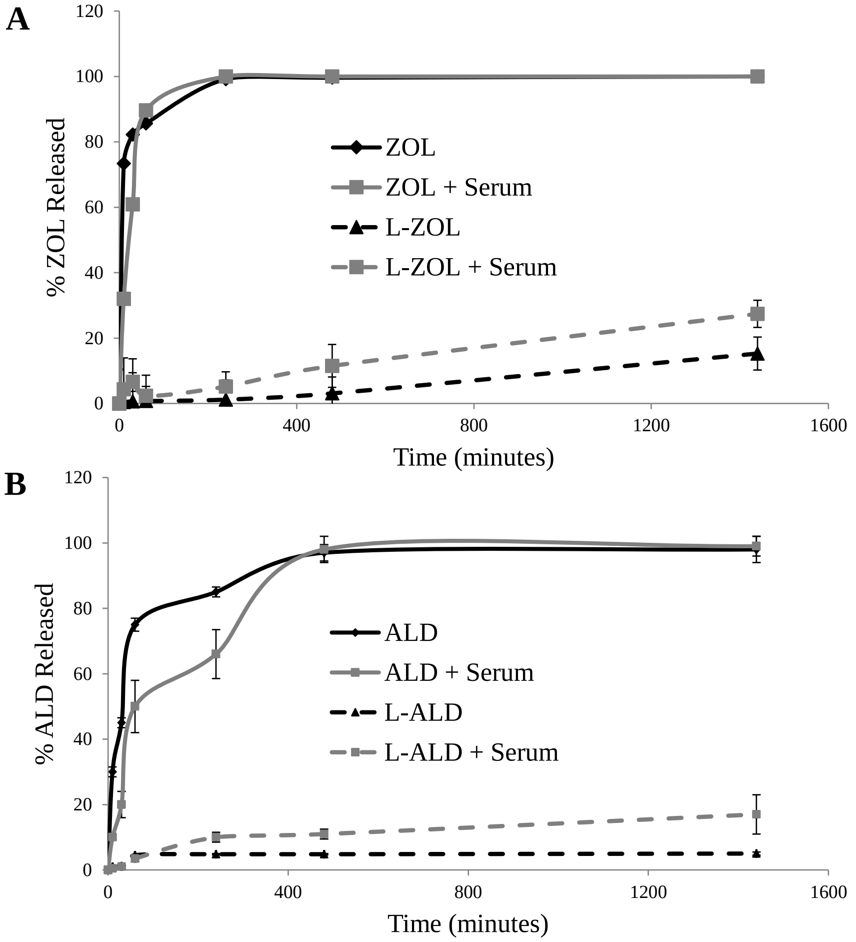

Figure S4: ZOL or ALD release from liposomes. The release profile of **(A)** ZOL liposomes and **(B)** ALD liposomes was determined in HBS or serum by dialysis. Liposomes were mixed with equal volumes of PBS or fetal bovine serum (FBS) and placed in a 10 kD MWCO dialysis bag. This was then placed in 200 ml HBS at 37°C with stirring to create sink conditions. Samples were taken at various time points up to 24 h. In the case of L-ZOL, samples were taken from inside the bag and measured with RP-HPLC with UV detection at 210 nm, while for L-ALD samples were taken from the external phase and measured using OPA method (SI). Release of 5-15 % and 17-27 % was seen at 24 h in HBS and FBS, respectively. Values are shown as mean ± SD (n=3).

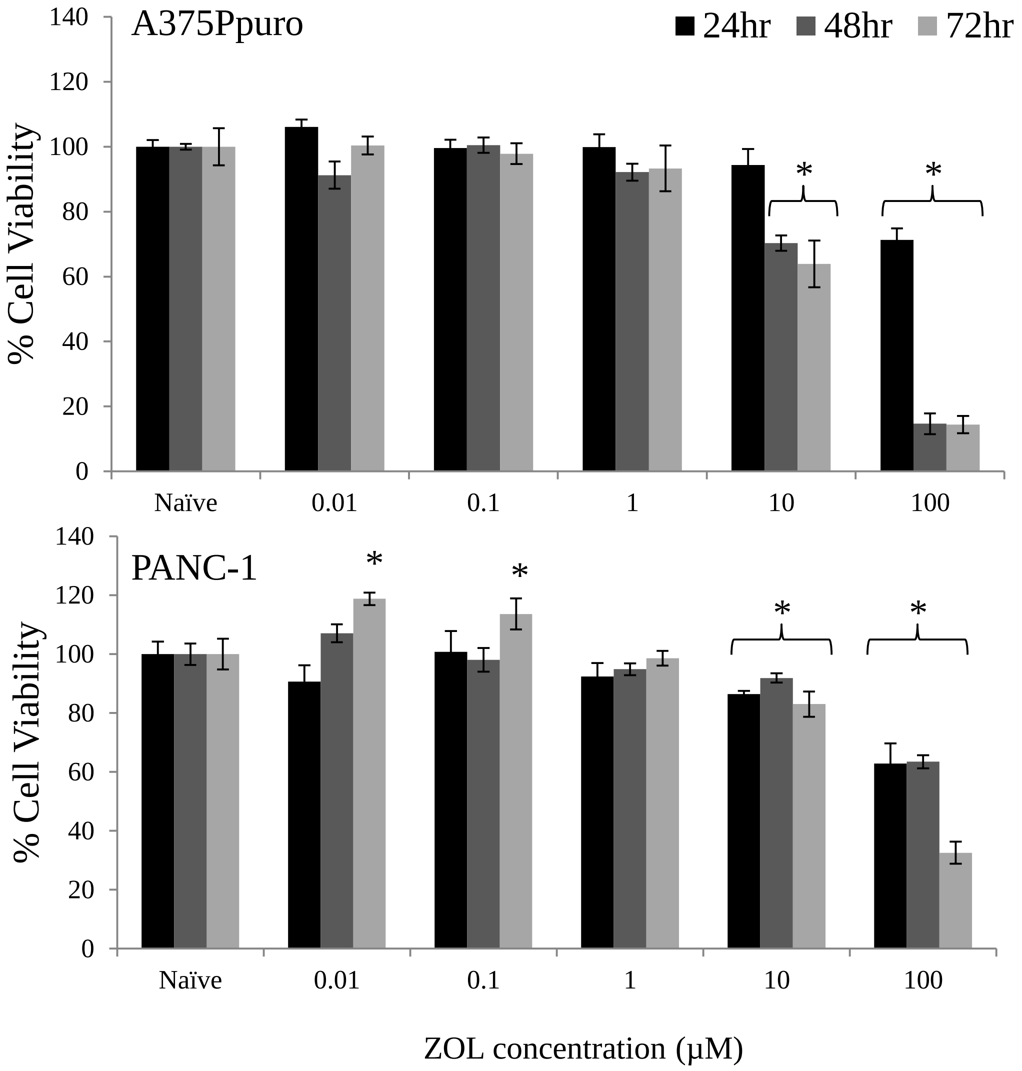

Figure S5: Cell viability of human cancer cell lines treated with ZOL. Percentage cell viability of two cancer cell lines after treatment with free ZOL. Cell viability was determined using MTT assay and values are presented as a percentage of untreated cells. Time- and dose-dependent toxicities were seen in both cell lines. ZOL concentrations used in subsequent γδ T-cell/ tumour cell co-culture studies were < 10 µM and ZOL was pre-incubated with cells for 24 h before addition of γδ T-cells. Data are expressed as mean ± SD (n=5). *$p < 0.05$, (Student's *t* test vs. naïve).

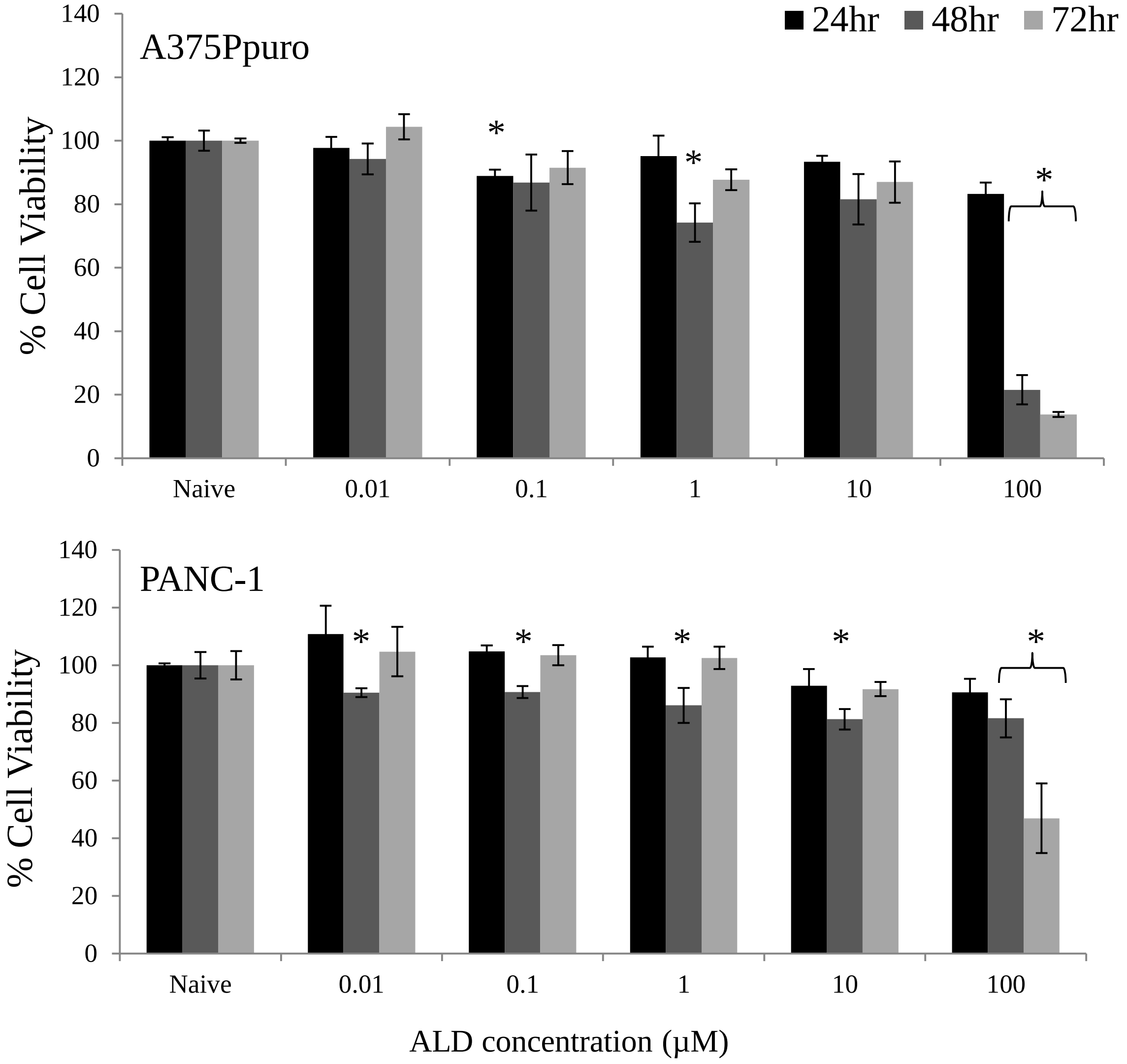

Figure S6: Cell viability of human cancer cell lines incubated with ALD. Percentage cell viability of the indicated two cancer cell lines after treatment with free ALD. Cell viability was determined using MTT assay and values are presented as a percentage of untreated cells. Time- and dose-dependent toxicities were seen in all cell lines. ALD concentrations used in subsequent γδ T-cell/ tumour cell co-culture studies were < 60 µM and ALD was pre-incubated with cells for 24 h before addition of γδ T-cells. Data are expressed as mean ± SD (n=5). *$p < 0.05$, (Student's *t* test vs. naïve).

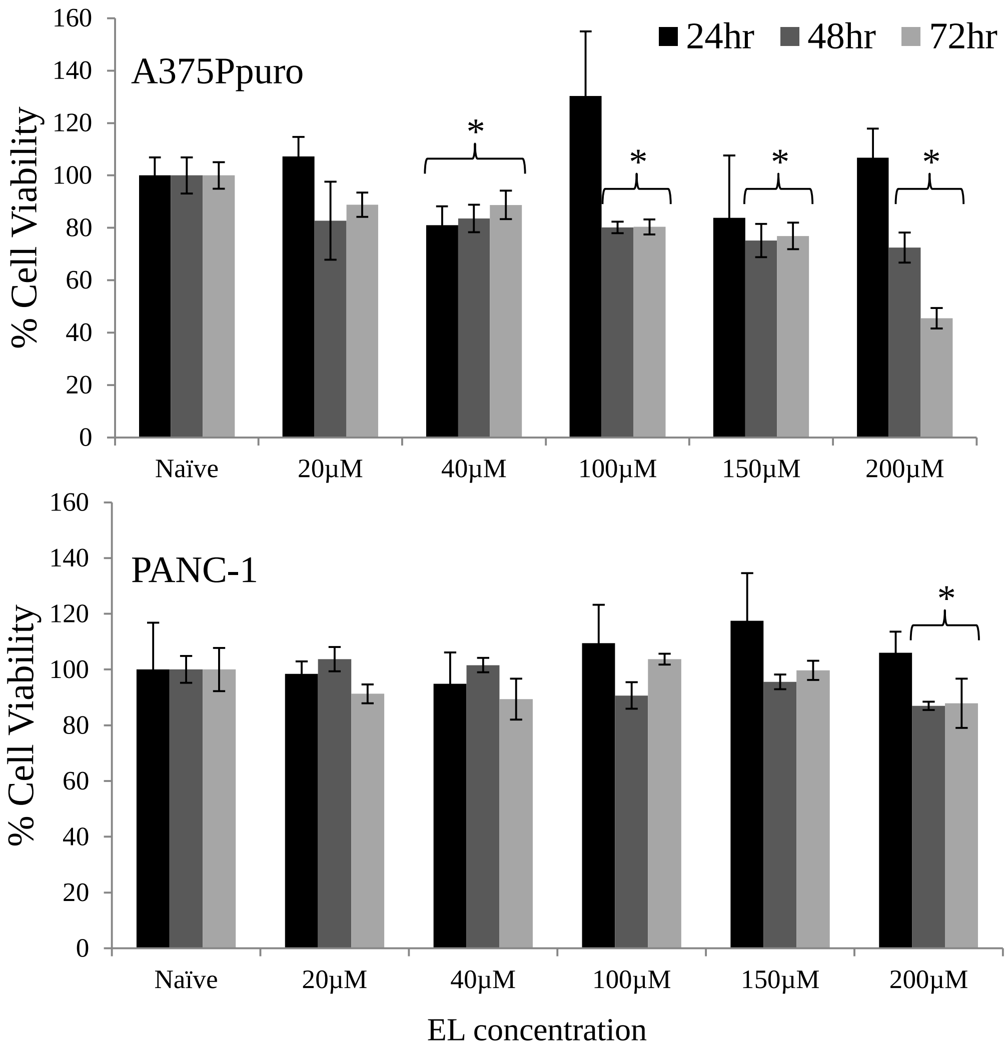

Figure S7: Cell viability of human cancer cell lines treated with empty liposomes. Percentage cell viability of the indicated two cancer cell lines after treatment with empty liposomes in. Cell viability was determined using MTT assay and values are presented as a percentage of untreated cells. Time- and dose-dependent toxicities were seen in A375Ppuro. Minimal cytotoxicity was seen in PANC-1. Lipid concentrations used in co-culture studies were equivalent to < 50 µM and < 200 µM (for 24h) in subsequent studies performed with liposomal formulations of ZOL and ALD, respectively. Data are expressed as mean ± SD (n=5). *p< 0.05, (Student's *t* test vs. naïve).

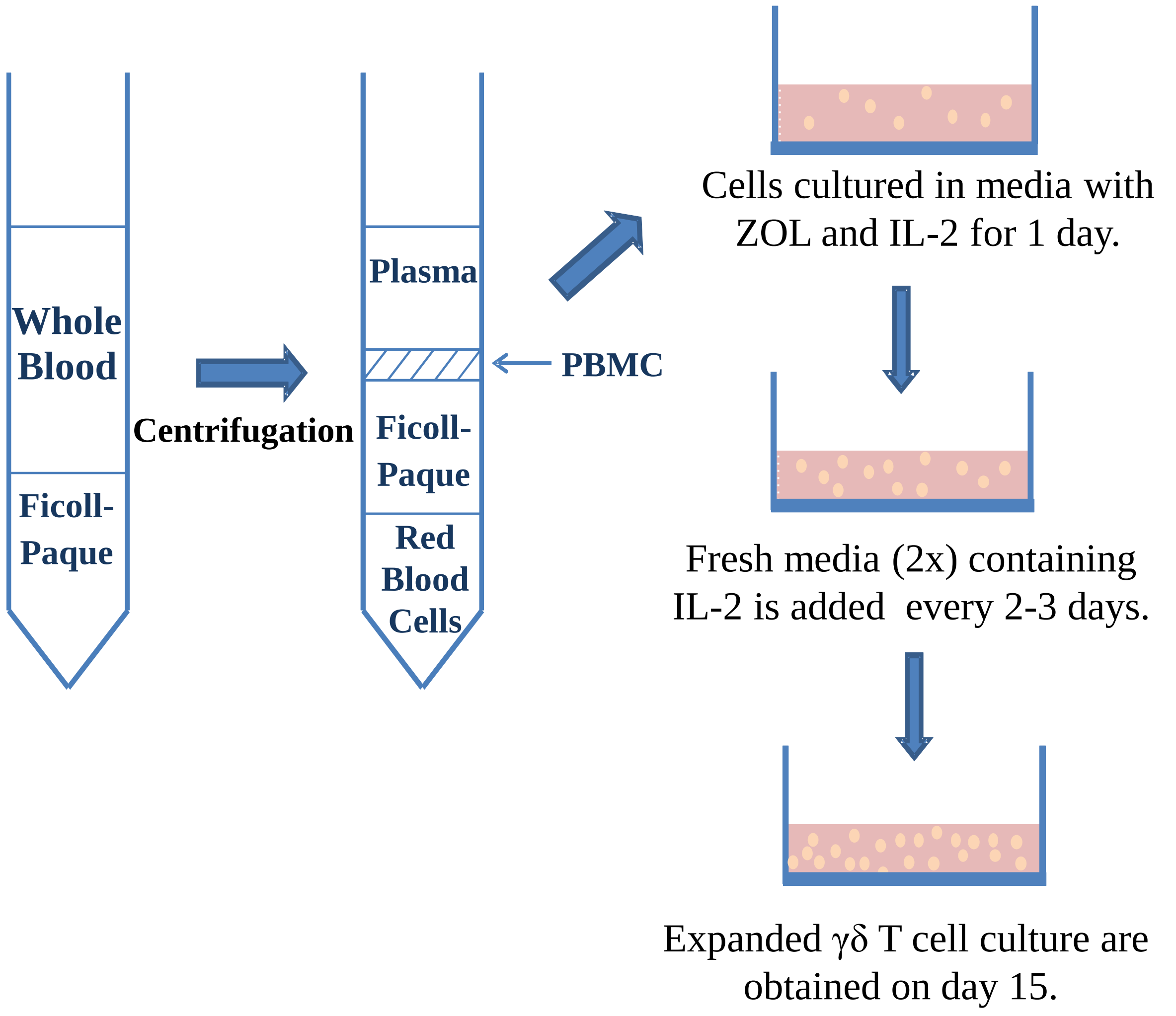

Figure S8: Scheme for isolation of γδ T cells from human whole blood samples. Blood samples were obtained from healthy volunteers. The blood sample was layered on top of Ficoll-Paque Plus and centrifuged in order to isolate the peripheral blood mononuclear cells (PRMCs). The PBMCs were washed in PBS and then re-suspended in complete media at a concentration of $3x10^6$ cells/ml. In order to preferentially expand the γδ T cells, the PBMCs were activated with 1 µg/ml ZOL and 100 U/ml IL-2. Additional medium and 100 U/ml IL-2 were added every 2-3 days for 15 days.

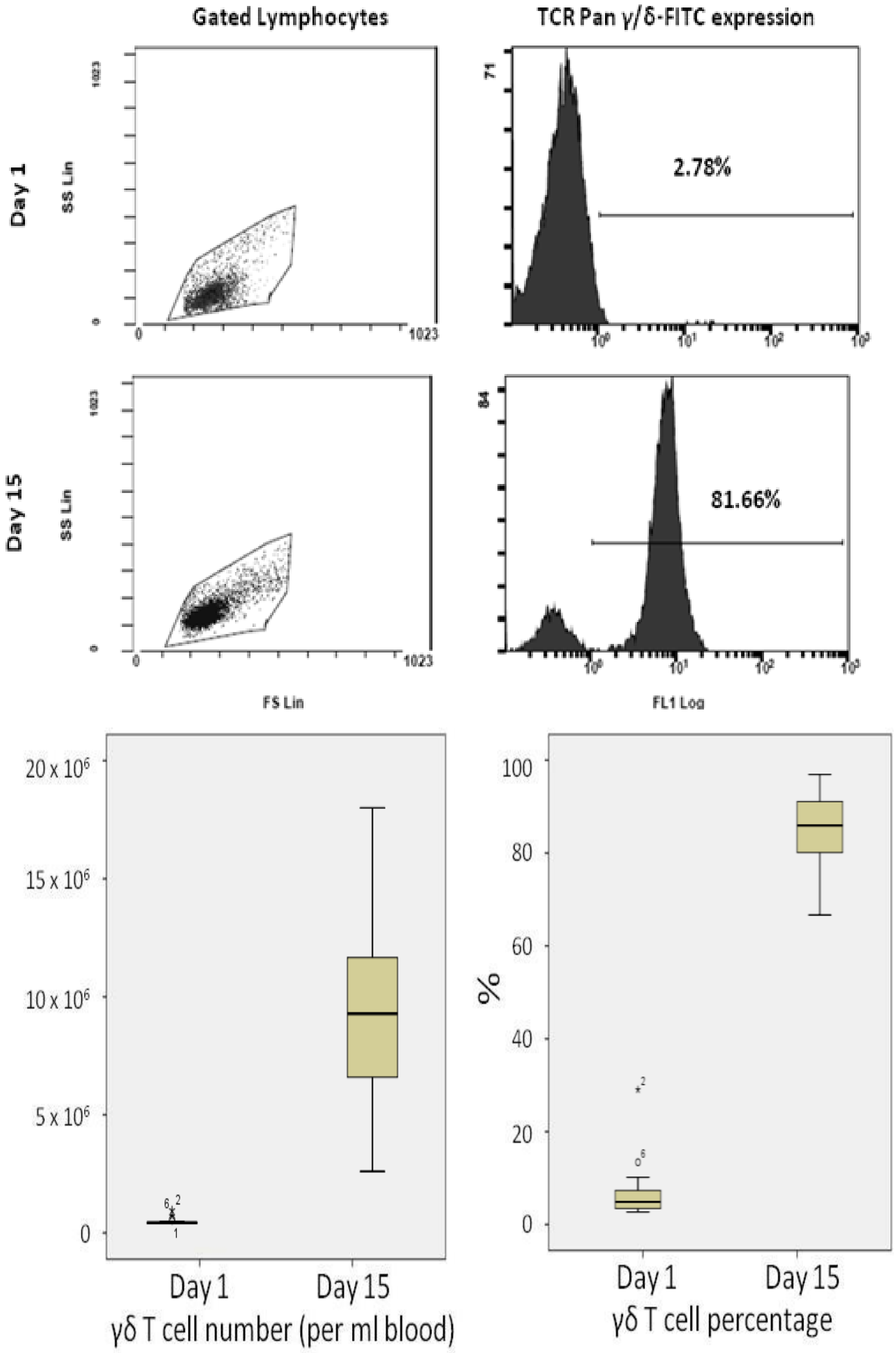

Figure S9: Characterisation of purity and yield of γδ T cells by flow cytometry. γδ T cells were isolated from 22 healthy donors. The number and percentage of γδ T cells were measured using the TCR Pan γ/δ-FITC antibody, with IgG1 FITC used as an isotype control. The cells were incubated with the antibodies for 20 min at 4°C, washed then re-suspended in PBS and analysed by flow cytometry. Percentage of γδ TCR positive cells were calculated as a percentage of the total lymphocytes. γδ T cell population was just under 5 % (ranging from 0.8 – 28.3 %) on day 1, and increased to 86 % on day 15. The number of γδ T cells expanded by 96 fold by day 15.

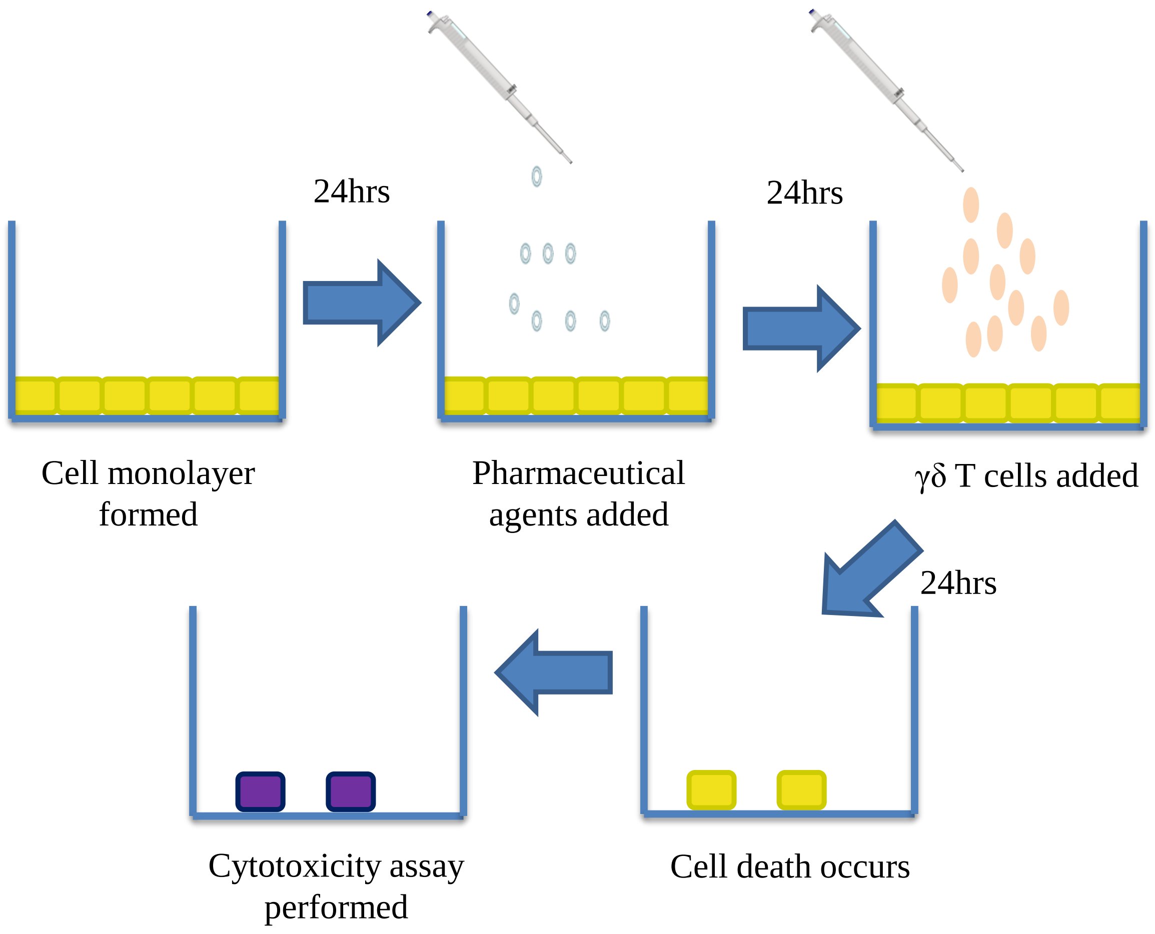

Figure S10: Scheme for co-culture studies of γδ T cells with human cancer cell lines. Confluent monolayers of each cancer cell line were treated for 24 h with ZOL, ALD, L-ZOL or L-ALD at various concentrations or were left untreated. After 24 h, the treatments were removed and the monolayers were then co-cultured with $2.5x10^5$ γδ T cells for another 24 h. An MTT assay was then performed to assess cell viability. MTT solution was prepared in PBS at a concentration of 5 mg/ml and was diluted in media (1:6) prior to use. The supernatant of each well was removed and MTT solution was added to each well. The plates were then incubated at 37°C for 3 h before the MTT solution was removed from each well and replaced with DMSO solubilise the crystals that had formed. The absorbance was then read at 570 nm with reference at 630 nm.

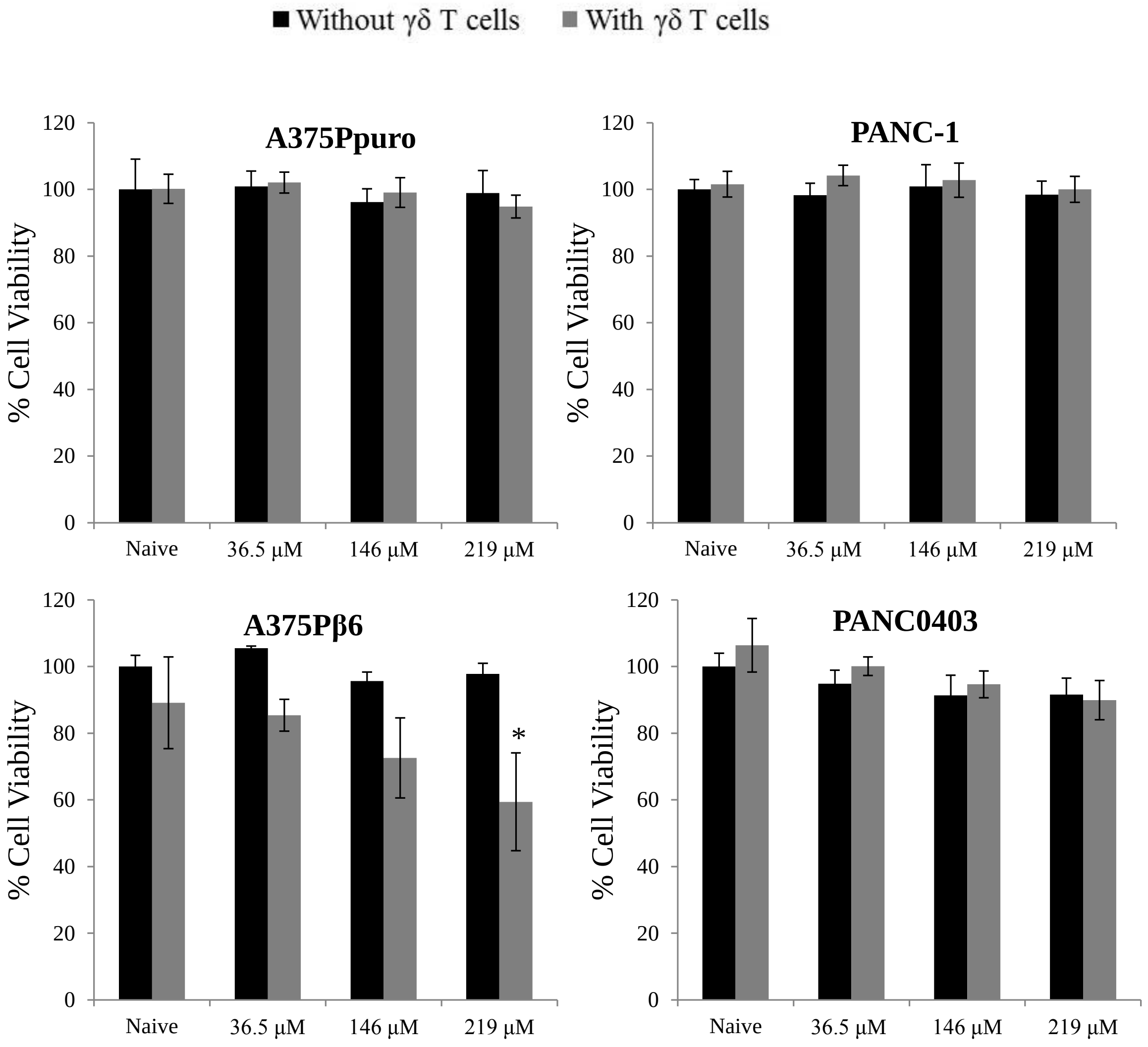

Figure S11: Cell viability of human cancer cell lines after incubation with γδ T cells and empty liposomes. A control experiment was performed to determine the background killing by γδ T cells. Confluent cell monolayers were treated with empty liposomes (36.5 – 219 µM) for 24 h before addition of γδ T cells (2 x 10^5). The co-culture was left for 24 h, followed by γδ T cells and cell washing prior MTT assay. These concentrations were chosen to reflect the amount of liposomes used when treating the cells with L-ZOL or L-ALD. No significant cytotoxicity was observed in the case of the empty liposomes alone or in combination with the γδ T cells, except for the cell line A375Pβ6 where a reduction in cell viability was seen at the highest concentration of liposomes used. Data are expressed as mean ± SD (n=5). $p < 0.05$, vs. (Student's *t* test vs. naïve).

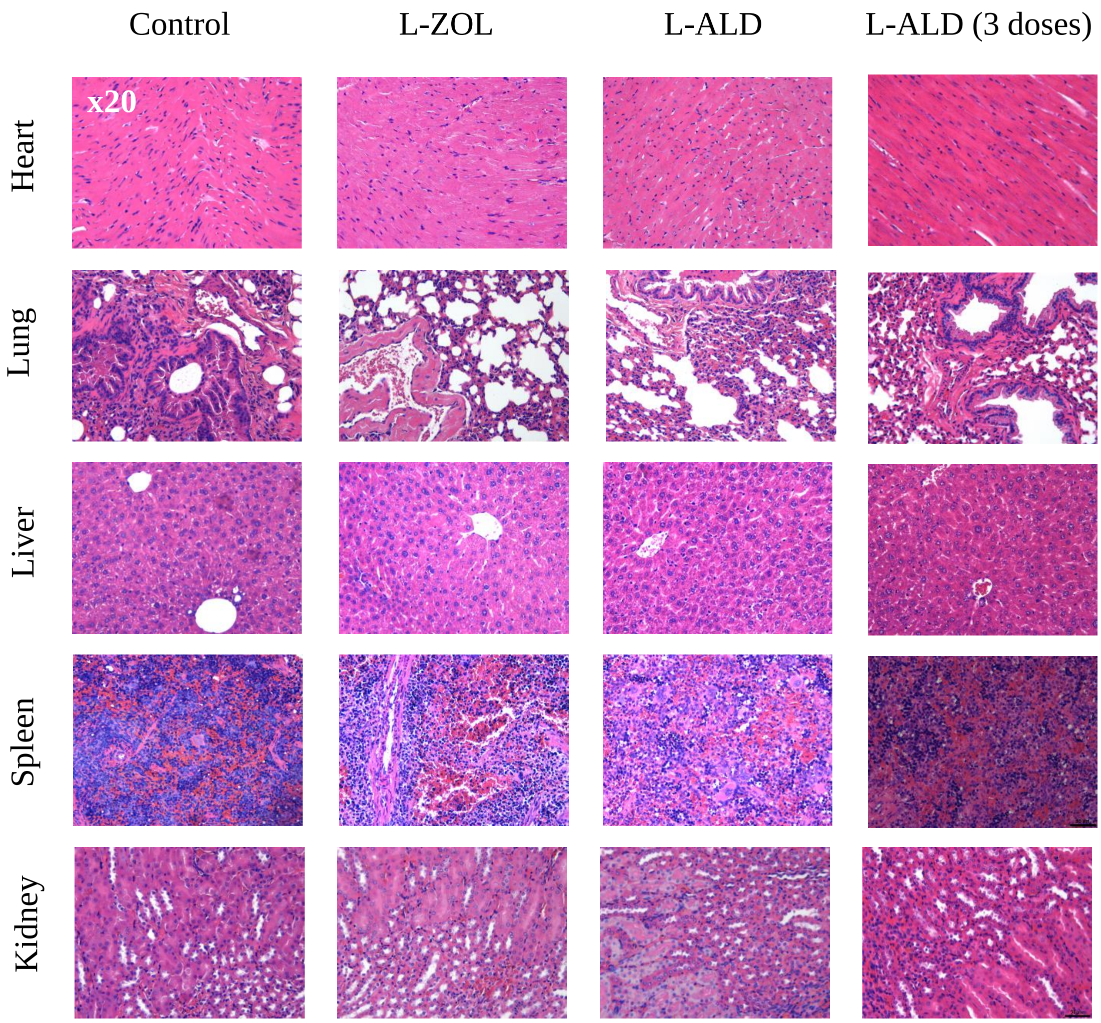

Figure S12: Effects of L-ZOL and L-ALD on organ histology. NSG mice were injected with 0.1 µmol L-ZOL or 0.5 µmol L-ALD. After 72 h, the mice were sacrificed and the organs were immediately fixed in 10% neutral buffer formalin as 5 mm^3 pieces. These pieces were then paraffin-embedded, sectioned for haematoxylin and eosin stains (H&E) and analysed by microscopy. No signs of toxicity were observed.

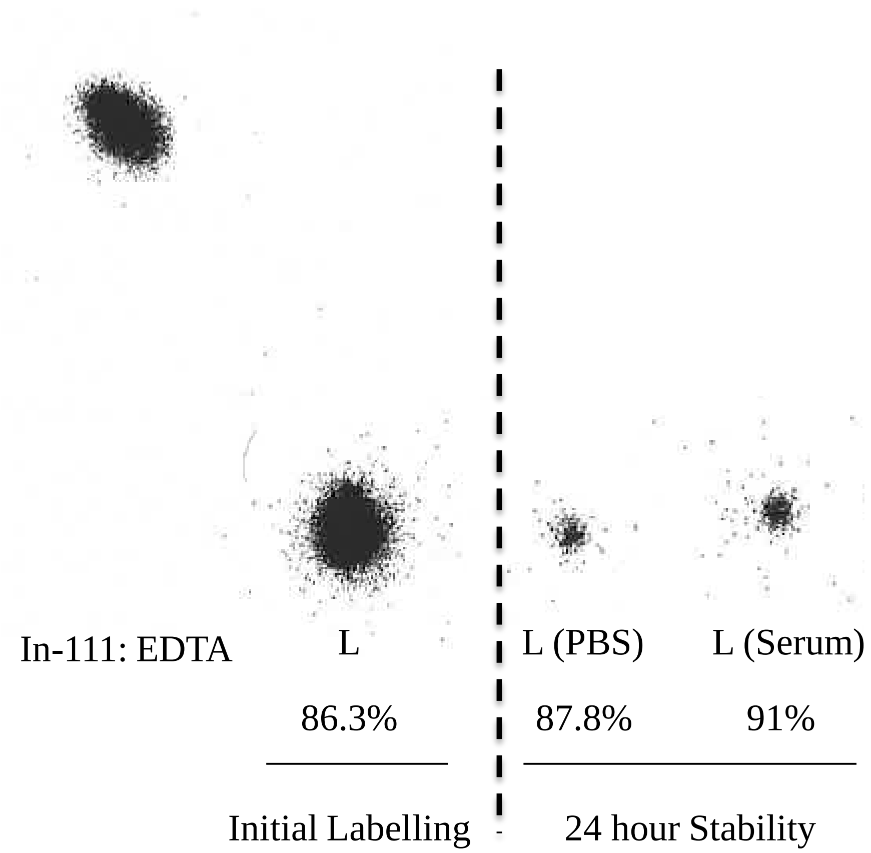

Figure S13: Radiolabelling of liposomes with ^{111}In and their stability in PBS or serum. TLC of the radiolabelled liposomes immediately after radiolabelling (left). Left: stability studies in FBS (50%) or PBS after 24 h incubation at 37°C.

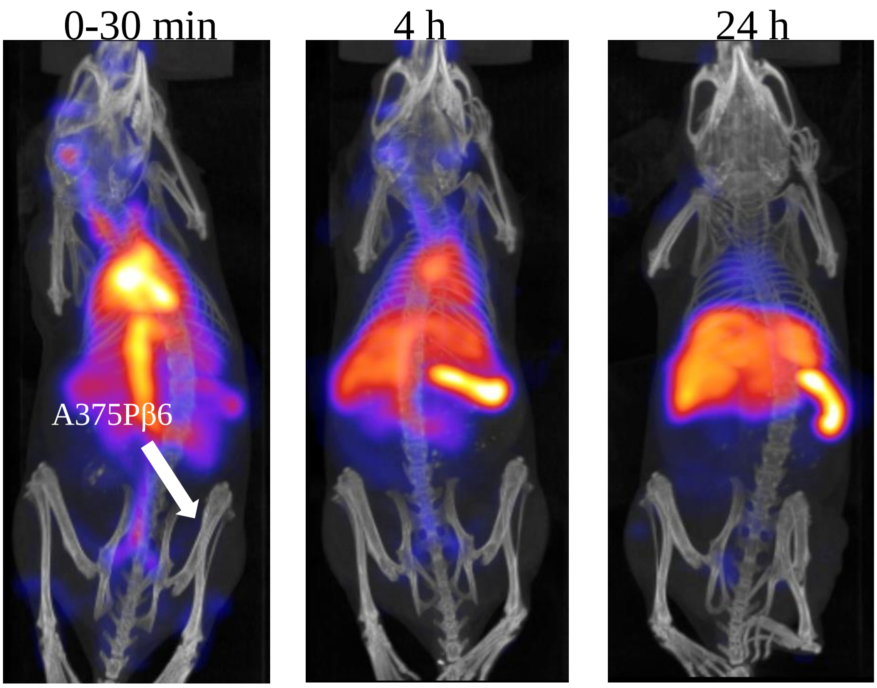

Figure S14: ***In vivo*** **biodistribution of radiolabelled EL, L-ALD and L-ZOL in A375Pβ6 tumour bearing NSG mice after single dose administration** ***via*** **tail vein injection.** NSG mice were inoculated bifocally with the A375Pβ6 cell line and were i.v. injected with ^{111}In labelled liposomes at a dose of 2 μmol lipid/mouse. Whole body SPECT/CT imaging was performed at 0-30min, 4 and 24 h post-injection of 2 μmol EL/mouse with scanning time of 40-60 min each.

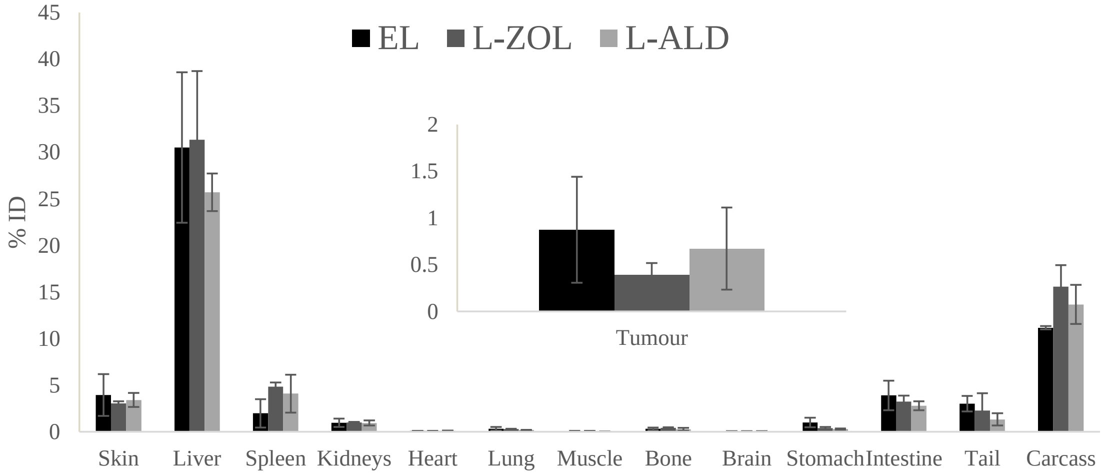

Figure S15: *In vivo* biodistribution of radiolabelled liposomes cells in tumour bearing NSG mice after single dose administration *via* tail vein injection. Mice were inoculated bifocally in each flank with the A375Pβ6 cell line and were i.v. injected with ^{111}In labelled liposomes at a dose of 2 μmol lipid/mouse. Results were expressed as percentage injected dose per organ (%ID) at 24 h after injection of 2 μmol liposome/mouse.
